# Efficacy and Tolerability of Cranial Electrotherapy Stimulation in the Treatment of Anxiety: A Systemic Review and Meta-Analysis

**DOI:** 10.3389/fpsyt.2022.899040

**Published:** 2022-06-09

**Authors:** Pao-Yuan Ching, Tien-Wei Hsu, Guan-Wei Chen, Chih-Chuan Pan, Che-Sheng Chu, Po-Han Chou

**Affiliations:** ^1^Department of Psychiatric, Kaohsiung Veterans General Hospital, Kaohsiung, Taiwan; ^2^Center for Geriatric and Gerontology, Kaohsiung Veterans General Hospital, Kaohsiung, Taiwan; ^3^Non-Invasive Neuromodulation Consortium for Mental Disorders, Society of Psychophysiology, Taipei, Taiwan; ^4^Graduate Institute of Medicine, College of Medicine, Kaohsiung Medical University, Kaohsiung, Taiwan; ^5^Department of Psychiatry, China Medical University Hsinchu Hospital, China Medical University, Hsinchu, Taiwan

**Keywords:** anxiety, cranial electrotherapy stimulation, depression, efficacy, meta-analysis

## Abstract

**Objective:**

We aimed to investigate the efficacy and tolerability of cranial electrotherapy stimulation (CES) for patients with anxiety symptoms.

**Method:**

We searched the Pubmed, Cochrane Central Register of Controlled Trials (CENTRAL), Embase and Medline for randomized control trials (RCTs) from the time of inception until November 15, 2021, following Preferred Reporting Items for Systematic Reviews and Meta-analyses guidelines. Data were pooled using a random-effects model. The primary outcomes were the mean change scores for anxiety symptoms. The secondary outcomes were the mean change scores for depressive symptoms.

**Results:**

Eleven RCTs were eligible (*n* = 794, mean age: 41.4, mean population of female: 64.8%). CES significantly reduced the anxiety symptoms compared to the control group [*k* = 11, *n* = 692, Hedge's g = −0.625, 95% confidence intervals (CIs) = −0.952 to −0.298, *P* < 0.001] with moderate effect size. The subgroup analysis showed that CES reduced both primary and secondary anxiety (primary anxiety, *k* =3, *n* = 288, Hedges' g = −1.218, 95% CIs = −1.418 to −0.968, *P* = 0.007; secondary anxiety, *k* = 8, *n* = 504, Hedges' g = −0.334, 95% CIs = −0.570 to −0.098, *P* = 0.006). After performing between group analysis, we found CES has significant better efficacy for patients with primary anxiety than those with secondary anxiety (*P* < 0.001). For secondary outcome, CES significantly reduced depressive symptoms in patients with anxiety disorders (*k* = 8, *n* = 552, Hedges' g = −0.648, 95% CIs = −1.062 to −0.234, *P* = 0.002). No severe side effects were reported and the most commonly reported adverse events were ear discomfort and ear pain.

**Conclusion:**

We found CES is effective in reducing anxiety symptoms with moderate effect size in patients with both primary and secondary anxiety. Furthermore, CES was well-tolerated and acceptable.

**Systematic Review Registration:** PROSPERO, https://www.crd.york.ac.uk/prospero/display_record.php?ID=CRD42021267916.

## Introduction

Anxiety disorders are the most common mental illness in the United States (US). It is estimated that about 31.1% of US adults had an anxiety experience at some time in their lives (1). Psychiatric comorbidity among patients with anxiety disorders are common. The epidemiology data shows 59.1% of generalized anxiety disorder (GAD) patients comorbid with major depressive disorder (MDD) during a 12-month period (2). Patients suffering from both anxiety and depression have greater impairment in occupational functioning, social functioning, and quality of life, thus leading to poor outcome and greater relapse rate (3, 4). Selective serotonin reuptake inhibitors (SSRIs) and cognitive-behavior therapy (CBT) are the most established treatments for anxiety disorders. However, the proportion of no-responders were up to one third for patients with anxiety disorder receiving either CBT or SSRIs due to poor compliance and adverse effects of medications (5).

Cranial electrical stimulation (CES) is one of non-invasive brain stimulation (NIBS) interventions and has been approved by the US Food and Drug Administration for the treatment of anxiety, depression, and insomnia (6). CES modulates brain function by applying pulsatile low-intensity current through earlobes or scalp (6). Transcranial direct current stimulation (tDCS) and repetitive transcranial magnetic stimulation (rTMS) were also commonly used as alternative treatment in psychiatric disorders (7, 8). tDCS and rTMS produced efficacy via giving direct current flow or pulsed magnetic field to the specific brain region (left dorsolateral prefrontal cortex most frequently targeted), inducing neuron excitatory or inhibitory effects (8). However, the tDCS and rTMS treatment have to be administered once-daily by a psychiatrist or well-trained specialists in a clinical environment. On the other hand, CES is a portable device and could be applied by patients alone at home. Therefore, CES is more accessible, time-saving, and affordable. Although the mechanism of CES is still unclear, a previous study has found that it may cause cortical deactivation and alter connectivity within the default mode network, one of main pathophysiological mechanisms for anxiety disorder (9, 10). In recent years, accumulating evidence suggests that CES may be an effective alternative treatment for anxiety disorders (11–13). For example, a randomized controlled trial (RCT) of 115 participants with anxiety disorder showed that the Hamilton Rating Scale for Anxiety (HAM-A) scores in the active CES group decrease more than three times than the sham CES group (11).

It is to our knowledge that only two meta-analyses to date have examined the potential effect of CES on treating anxiety symptoms (14, 15). One meta-analysis of eight RCTs showed CES were significantly more effective than controlled group. However, the quality and quantity of included trials were poor (15). Another meta-analysis which contained 26 RCTs was published in 2018 (14). Six of the included RCTs measured anxiety symptoms as outcome, and the result of the study showed that CES has modest benefit in patients with anxiety symptoms. Furthermore, five out of six trials included in the study were published 20 years ago (10, 11, 16–19). After the United States Food and Drug Administration first approved CES devices for medical treatment in 1978, updated CES devices with enhanced technique have been marketed such as Alpha-Stim SCS, Alpha-Stim 100 and CMS generator (11, 20–22). The distinct ability of newer devices provided more steady stimulation current, less variability of function, and thus more controllable effectiveness (23). In addition, several new double blinded RCTs have been published in more recent years (21, 22, 24–26). The design of double-blinded, sham controlled trials can eliminate the placebo effects both generated by the investigators and the subjects, minimizing the methodological heterogeneity that are commonly observed in the CES trials (20, 27).

Therefore, the current systematic review and meta-analysis study aims to reappraise available evidence to investigate the efficacy of CES on anxiety symptoms. In addition, we examined whether there is different efficacy of CES in reducing anxiety symptoms among patients with primary anxiety disorder or secondary-caused anxiety symptoms that might be attributed to other medical condition.

## Methods

### Database Searches

This study was conducted and reported per the Preferred Reporting Items for Systematic Reviews and Meta-Analyses guidelines (PRISMA) (28). The PICO (population, intervention, comparison, and outcome) settings of the current meta-analysis were (1) population: patients with anxiety disorders; (2) intervention: cranial electrotherapy stimulation; (3) comparison: a control therapy; (4) outcome: changes in anxiety symptoms. Pubmed, Cochrane Central Register of Controlled Trials (CENTRAL), Embase and Medline were systematically searched from the time of their inception until November 15, 2021. The search term we use were (CS OR Cranial Electrotherapy Stimulation OR cranial electrotherapy stimulation OR non-invasive brain stimulation OR Alpha-Stim) AND (anxiety OR anxious OR panic OR phobia OR worrisome OR insomnia OR sleep OR depression OR suicide) without any limitation on language. The reference lists of included articles and recent reviews were also searched to identify additional references.

### Eligible Criteria and Study Selection

The following eligible criteria were applied: (i) peer-reviewed original articles of RCTs investigating the effects of CES as monotherapy or combination with other treatment (e.g., biofeedback therapy and antidepressants) for management of anxiety symptoms; (ii) diagnosis of anxiety disorders include general anxiety disorder (GAD), panic disorder, mixed anxiety disorder, specific phobia, social phobia, agoraphobia, and other anxiety diseases which meet the criteria in DSM-IV, DSM-IV TR, DSM-V or ICD10 (29–32); (iii) definition of anxiety symptoms based on screening tool; (iv) a comparison between an intervention group and a control group (e.g., biofeedback therapy, antidepressants); (v) sufficient data for both the intervention group and the control group; (vi) articles written in English. We excluded non-clinical trials such as case series or observational studies. Conference abstracts and studies published in languages other than English were also excluded.

### Methodological Quality Assessment

Two authors (TW Hsu and CS Chu) independently assessed the methodological quality of the included studies using The Jadad score (33) and the Cochrane Risk of Bias version 2 (RoB2) (34). The Jaded score consists of a five-point questionnaire, ranging from zero (poor quality) to five (high quality), which is used to assess the studies in three categories: randomization, withdrawals and dropouts, and blindness. In case of discrepancies, another author (PY Ching) was consulted to obtain a consensus.

### Data Extraction

The data of included studies were extracted by two of the authors (PY Ching and TW Hsu) in accordance with a pre-specified data extraction form independently. Any discrepancies in the inclusion between the reviewers were resolved by the third investigator (CS Chu).

A pre-specified data extraction form was used to extract data for this meta-analysis. The data extracted from studies consist of basic characteristics of participants (mean age, percentage of female), study quality measured using the Jadad scoring system and the protocol of CES (frequency, the strength of current, duration of each session, total treatment sessions).

### Primary and Secondary Outcomes

We defined the primary outcomes as mean change in scores for anxiety symptoms. The anxiety symptoms had to be assessed by using a validated scale such as Hamilton Anxiety Rating Scale (HAM-A) (35), Zung Self-Rating Anxiety Scale (SAS) (36), Hospital Anxiety and Depression Scale (HADS) (37), Profile of Mood States (POMS) (38), the Korean edition of the Profile of Mood States (K-POMS) (39), State-Trait Anxiety Inventory (STAI) (40), Visual Analog Scale for Anxiety (VAS-A) (41). Among recruited studies, only one study used two scales at the same time to assess anxiety outcome (42). We used the VAS-A scales because it provides adequate raw data and is most frequently used scales.

Any unavailable data were recorded as missing data.

The secondary outcome was defined as depression symptoms, which were obtained by data from each study. The Hamilton Depression Rating Scale (HAM-D) (43), Center for Epidemiologic Studies Depression Scale (CES-D) (44, 45) and Zung Self-Rating Depression Scale (SDS) (44) were the most frequent used scale to assess depression. The depression symptoms had to be measured by screen tools including the SDS (44), HAM-D (43), CES-D (45), HADS (37), and POMS (38).

### Meta-Analysis Procedure

Owing to an anticipated heterogeneity across included studies, we conducted a random-effect meta-analysis (46). We calculated Hedges'g statistic for the estimation of within-group effect size (ES) and 95% Confidence intervals (CIs) as changes from pre-treatment to post-treatment and between-group (intervention group vs. control group) effect size for the primary outcome. When we need to assess different scales in each trial, standardized mean differences (SMD) were calculated for each trial and used to derive total estimates on the outcomes (47). Standard error or *t*-value was used to estimate those trials without data of standard deviation. Regarding the handle of the SD of the change scores, we imputed a change-from baseline SD using a correlation coefficient of 0.5 based on Cochrane Handbook for Systemic Reviews of Intervention (48). For interpretation of effect sizes, we followed the rules of classifying <0.2 as very small, 0.2–0.5 as small, 0.5–0.8 as moderate and >0.8 as large (46). Odd ratios (ORs) and 95% CIs were calculated for dichotomous data. All meta-analytic procedures were performed using Comprehensive Meta-Analysis software, version 3 (Biostat, Englewood, NJ, United States). The threshold for statistical significance was set at a two-tailed *P*-Value of <0.05.

### Sensitivity Analysis, Heterogeneity, Publication Bias, Meta-Regression Analyses, and Subgroup Analysis

Heterogeneity was assessed using the Cochran's *Q* test and the I2 metric. Publication bias was assessed via the inspection of funnel plots and using the Egger's regression test. Meta-regression analyses were conducted with unrestricted maximum likelihood random effects when data on each potential moderator were used in at least five different studies (49). Percentage of female gender, and Jadad scores, stimulation protocol (frequency, the strength of current, duration of each session, and total treatment sessions) were considered as variables for meta-regression. Primary anxiety disorder such as GAD, panic disorder, mixed anxiety disorder, specific phobia, social phobia, and agoraphobia is different from secondary anxiety disorder caused by a medical condition. Secondary anxiety (usually comorbid with general medical conditions) was associated with poorer outcomes than primary anxiety disorder. Therefore, we conducted subgroup analysis to explore whether efficacy of CES will differ between primary anxiety disorder and secondary caused anxiety symptoms.

## Results

### Studies in the Meta-Analysis

**Figure 1:**
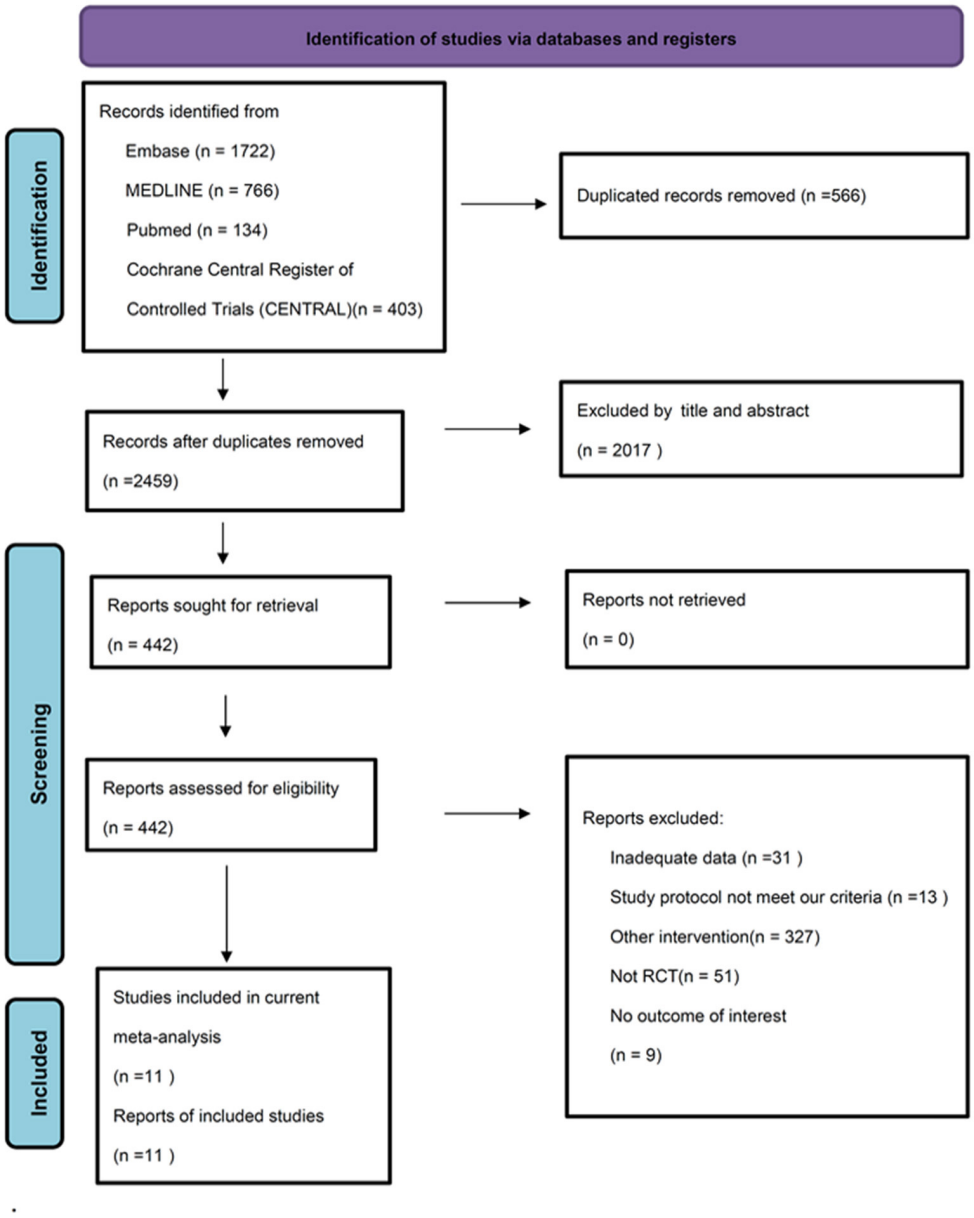
PRISMA flowchart of study selection.

We identified 2,459 potential articles after searching the database and removed duplicate records. Among these articles, we excluded 2017 of them by screening title and abstract. In addition, 431 studies were excluded through full-texted assessment with specific reasons (Supplementary Table S2). Ultimately, 11 studies were included (Table 1) (11–13, 21, 22, 24, 26, 42, 50–52). The flowchart of our search strategy is presented in Figure 1. We included 794 participants (mean age, 41.4 +/– 8.7; female, 64.8%). All these 11 trials are RCTs comparing CES with control/sham group. Eight of them used sham stimulation (11, 13, 21, 24, 26, 42, 51, 52) and three of them used active control including aerobic exercise (50), biofeedback (22) and paroxetine (12). Three studies used home-based CES (11, 26, 52), four studies applied CES in the hospital (13, 22, 24, 50) and unclear information was provided in the remaining three studies (12, 21, 51). All 11 RCTs provided data for analysis of anxiety severity as primary outcome, whereas eight of them provided data for analysis of depressive symptoms as secondary outcome.

**Table 1:** The characteristics and demographics of the included studies.

**Reference**	**Country**	**Population**	**Study design**	**Settings**	**Patient, *n***	**Total sessions**	**Session duration**	**Age (intervention, control)**	**Female % (intervention, control)**	**Outcome scale**
Do (21)	Korea	Patients with tension-type headaches, outpatient	Double blinded sham controlled, RCT	Intervention group: CMS generator, 25 mA, 8 Hz	Intervention group: 12 Control group (sham): 12	14 sessions	20 min	62.9 (all)	90.90 (all)	HADS
Wu (24)	China	Tic disorders in children and adolescents, inpatient	Double-blind, sham-controlled, RCT	Intervention group: CES ultra-stimulator, 500 μA−2 mA, 0.5 Hz	Intervention group: 29 Control group (sham): 24	40 sessions	30 min	11.31, 10.28	20.8, 15.1	HAM-A
Cho (50)	Korea	Obese middle-aged women, outpatient	RCT	Intervention group: Alpha-Stim 100, 100 mA, 0.5 Hz and aerobic exercise (CES+EX)	Intervention group: 12 Control group (aerobic exercise): 12	24 sessions	20 min	54.75, 54.83	100, 100	K-POMS
Gong (22)	China	Patient with functional constipation, outpatient	RCT	Intervention group: Alpha-Stim SCS, 10~500 mA, 0.5 Hz and Biofeedback therapy	Intervention group: 38 Control group (biofeedback therapy): 36	30 sessions	30 min	53.5, 53.2	65.8, 85.6	SAS SDS
Lyon (26)	United States	women receiving chemotherapy for early-stage breast cancer, inpatient	Sham controlled, RCT	Intervention group: Alpha-Stim 100, 100 mA, 0.5 Hz	Intervention group: 70 Control group (sham): 67	126 sessions	60 min	51.5 (all)	100 (all)	HADS
Barclay (11)	United States	Anxiety disorder, outpatient	Double-blind, sham controlled, RCT	Intervention group: Alpha-Stim 100, 100 mA, 0.5 Hz	Intervention group: 57 Control group (sham): 51	35 sessions	60 min	42.3 (all)	47.4, 52.6	HAM-A HAM-D
Lu (12)	China	Anxiety disorder, outpatient	RCT	Intervention group: Alpha-Stim SCS, 10~500 mA, 0.5 Hz and 10–20 mg/day of paroxetine	Intervention group: 60 Control group (paroxetine 10–20 mg/day): 60	42 sessions	60 min	32.6, 31.1	60, 66.7	HAM-A
NCT00723008	United States	Post-traumatic stress disorder (PTSD) in the burn patient, outpatient	Sham controlled, RCT	Intervention group: Alpha Stim 100, 100 mA, 0.5 Hz	Intervention group: 11 Control group (sham): 9	20 sessions	60 min	26.8 (all)	45 (all)	VAS CES-D
Tan (52)	United States	Spinal cord injury, inpatient	Double-blind, sham-controlled, RCT	Intervention group: Alpha-Stim SCS, 100 mA, 0.5 Hz	Intervention group: 45 Control group (sham): 55	21 sessions	60 min	53.1, 52.5	14.20 (all)	STAI CES-D
Chen (13)	China	mixed anxiety and depressive disorder, outpatient	sham controlled, RCT	Intervention group: Alpha Stim 100, 100~500 muA, 0.5 Hz	Intervention group: 30 Control group (sham): 30	15 sessions	10~15 min	12, 11	‘16.7, 36.7	SAS SDS
Cork (51)	United States	Patient with fibromyalgia	Double blinded sham controlled, RCT	Intervention group: Alpha-Stim CES, 100 mA, 0.5 Hz	Intervention group: 39 Control group (sham): 35	21 sessions	60 min	53 (all)	94.6 (all)	POMS

*The characteristics and demographics of the included studies*.

*RCT, randomized-controlled trial; HAM-A, Hamilton anxiety depression rating score; HAM-D, Hamilton depression rating score; K-POMS, Korean edition of profile of mood states; POMS, the profile of mood states; HADS, Hospital anxiety and depression scale; SAS, self-rating anxiety scale; SDS, self-rating depression scale; VAS, visual analog scale; CES-D, the center for epidemiologic studies depression scale; STAI, the state-trait anxiety inventory*.

### Methodological Quality of the Included Studies

We used the Jadad score system (33) and RoB2 (34) to assess the quality of the included studies. Across all 11 RCTs, the average Jadad score was 3.19 (Supplementary Table S3). Five studies were judged as having some concerns risk of bias and others as having low risk based on the Cochrane's RoB2 criteria (Supplementary Figure S4).

### Primary Outcome: The Effect of CES on the Anxiety Symptoms

**Figure 2:**
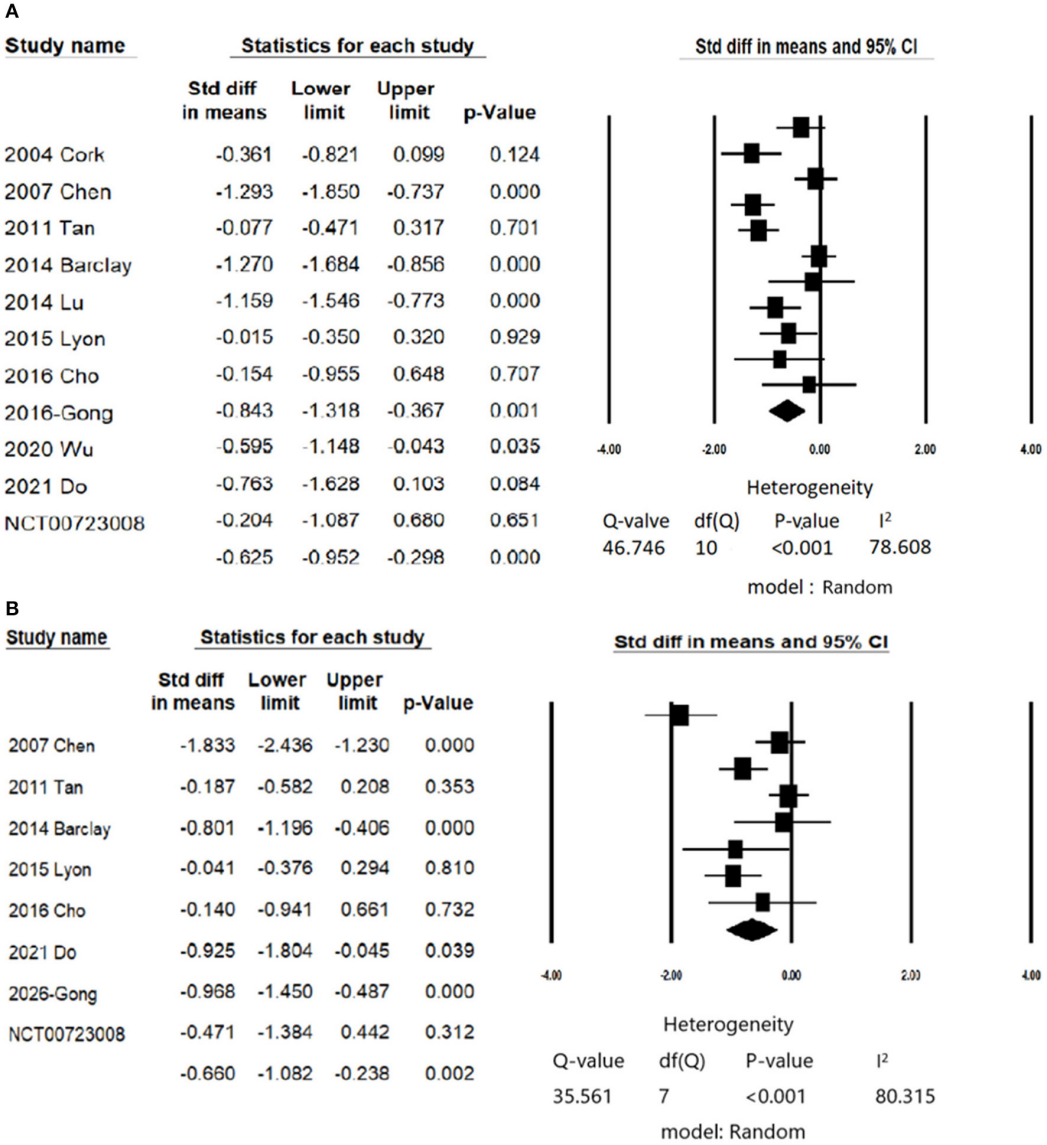
Forest plot of meta-analysis of **(A)** Primary outcome: change in scores of anxiety symptoms; **(B)** Secondary outcome: change in scores of depression symptoms.

In patients with anxiety symptoms, CES significantly improved anxiety symptoms with moderate effect size (anxiety symptoms, number of trials = 11, *n* = 692, Hedge's g = −0.625, 95% CIs = −0.952 to −0.298, *P* < 0.001 (Figure 2A) compared than control/sham group. There was no evidence of publication bias (Egger's regression test, *t* = 0.242, *P* = 0.81), but significant heterogeneity was found (*Q* value = 46.7 *I*^2^ = 78.6%, *P* < 0.001). The female percentage, Jadad score, treatment protocols (total sessions and duration of each session) did not contribute to the heterogeneity (Supplementary Table S5A).

### Secondary Outcome: The Effect of CES on the Depressive Symptoms

Eight studies examined the efficacy of CES on depressive symptoms (11, 13, 21, 22, 26, 42, 50, 52). CES significantly reduced depressive symptoms in patients with anxiety disorders (number of trials = 8, *n* = 552, Hedges' g = −0.648, 95% CIs = −1.062 to −0.234, *P* = 0.002; Figure 2B) compared than control/sham group. There was no evidence of publication bias (Egger's regression test, *t* = 0.9955, *P* = 0.357), but significant heterogeneity was found (*Q* value = 35.56 *I*^2^ = 80.31, *P* < 0.001).

### Sources of Heterogeneity of Subgroup Analysis

We conducted subgroup analysis examining the efficacy of CES on anxiety for patients with primary anxiety and secondary anxiety. CES treatment significantly reduced anxiety symptoms both in patients with primary anxiety and secondary anxiety (primary anxiety: number of trials =3, *n* = 288, Hedges' g = −1.218, 95% CIs = −1.418 to −0.968, *P* = 0.007; secondary anxiety, number of trials = 8, *n* = 504, Hedges' g = −0.334, 95% CIs = −0.570 to −0.098, *P* = 0.006; Table 2); furthermore, the CES showed greater effect on reducing anxiety symptoms among patients with primary anxiety than those with secondary anxiety (between group analysis, *P* < 0.001). The female percentage, Jadad score, treatment protocols (total sessions and duration of each session) did not contribute to the heterogeneity (Supplementary Table S5B). As for the depressive symptoms, CES has showed treatment efficacy in the group of secondary anxiety patients (number of trials = 6, *n* = 379, Hedges' g = −0.401, CIs = −0.750 to −0.053, *P* = 0.024). However, the analysis in primary anxiety group could not be performed due to lack of three studies available (Table 2).

**Table 2:** Subgroup analyses of CES on anxiety and depressive symptoms divided by primary and secondary anxiety.

	**Improvement in anxiety symptoms scale (Hedges' g, 95% CI)**	**Improvement in depressive symptoms scale (Hedges' g, 95% CI)**
**Diagnoses**	
Primary anxiety	−1.218 (−1.468 to −0.968) *P* < 0.001, *k* = 3, *n* = 288	NA, *k* < 3
Secondary anxiety	−0.334 (−0.570 to −0.098) *P* = 0.006, *k* = 8, *n* = 504	−0.401 (−0.750 to −0.053) *P* = 0.024, *k* = 6, *n* = 379

*CES, cranial electrotherapy stimulation; CI, confidence interval; k, number of trials; NA, not available*.

### Risk of Bias, Adverse Effects, and Attrition

Based on the Cochrane RoB2 criteria, five of the overall studies were judged as having some concern of bias. Risk for some concern ROB in each domain ranged from 0 to 38.7%. One study (42) was judged as having high bias in missing outcome data.

There were five studies reporting the numbers of adverse events (11, 13, 26, 42, 52) and no severe adverse events were reported. The most commonly reported adverse events were ear discomfort and ear pain (*n* = 41).

Ten studies reported attrition numbers. There was no significant difference between CES intervention group and control group regarding numbers of drop out (odd ratio = 0.699, 95% CIs = 0.430–1.136 *P* = 0.148 (Supplementary Table S6 and Supplementary Figure S7).

## Discussion

Our study used comprehensive meta-analysis that involved data from 11 RCTs to assess efficacy and tolerability of CES on anxiety symptoms among patients with anxiety disorders. We summarized our findings as follows: First, CES significantly reduced anxiety symptoms with moderate effect size compared to control group. Second, CES significantly reduced anxiety symptoms in patients with primary and secondary anxiety; furthermore, CES provided greater efficacy in those with primary anxiety. Third, CES significantly reduced depressive symptoms in patient with anxiety symptoms. Finally, no significant differences regarding attrition and adverse event in both group; therefore, CES was well-tolerated and acceptable.

The main findings of present study were consistent with prior studies, showing CES provided moderate effect on reducing anxiety (14). Furthermore, the present study provided additional advantages compared to previous studies. First, the present study enrolled more studies with 11 RCTs compared to previous study conducted by Shekella et al. (14) (*k* = 6). Therefore, the findings of the present study minimize the methodological heterogeneity (20, 27) due to the nature of double blinded RCT study design; Second, majority of studies included in the present work used new CES devices, called Alpha-Stim products FDA-approved in the United States. Alpha-Stim provided a patented waveform and had been increasing widely used across the United States. Therefore, the findings could provide further evidence in the real-world practice; third, we conducted subgroup analysis according to primary and secondary anxiety. Taken together, the present meta-analysis study confirmed the efficacy of CES on reducing anxiety symptoms. Furthermore, patients receiving CES reported mild adverse events with ears discomfort as most common symptoms. Therefore, CES could be considered as an effective and well-tolerated treatment for patients suffering from anxiety.

Another interesting finding of the present study was we confirmed the efficacy of CES on both primary and secondary anxiety. It is not uncommon for patients with physical conditions comorbid with anxiety. The recruited eight RCTs for secondary anxiety included patients with fibromyalgia (51), obesity (50), tension-type headache (21), breast cancer (26), burned trauma (42), chronic pain (52), constipation (22), and Tourette disorder (TD) (24). Among them, five RCTs found CES not only reduced anxiety but also significant improved primary symptoms such as pain (51), constipation (22), and symptoms of TD (24). To treat anxiety among patients with physical comorbidities is vital because literatures showing the poor disease outcome, increasing need for care and social burden, and decreased quality of life in such population (53, 54). CES might be a considerable alternative treatment, especially it reduced the drug-drug interaction and possible side effect derived from psychotropic agents.

The mechanisms underlying the effect of CES on reducing anxiety and/or depression remain elusive. The most recent review article proposed that the mechanism of CES was through affecting brain activity, neurotransmitter and hormone response (20). The computational modeling also demonstrated that the current produce by CES can reach cortical and subcortical region, thus affecting neural functioning (20). Another imaging study of brain MRI showed CES stimulation caused brain deactivation and alteration of default mode network (10). In summary, the efficacy of CES on reducing psychiatric symptoms might through several different mechanisms. Future studies are warranted to examine the underlying mechanism of CES.

### Limitation

Several limitations should be addressed. First, 56.6% (6/11) of overall RoB2 was ranked as low risk of bias, although 45.4% (5/11) of overall RoB2 was ranked as some concerns risk. Second, the included studies conducted different stimulation protocol (frequency, total sessions, current flow etc.); therefore, it is hard to conclude the most effective stimulation strategy. However, majority of studies used 0.5 Hz, 60 min per session, daily stimulation, which was considered as acceptable protocol (6). Third, the present study only analyzed the acute treatment effect of CES on anxiety. The long-term efficacy of CES on anxiety is unclear due to the lack of available data. Fourth, the placebo effect is critical issue in the RCTs, particular for brain stimulation (55), although Barclay et al. reported 28% overall changes for anxiety scores in sham group from baseline is within the limit of placebo responses (11, 56). Fifth, the efficacy of CES on insomnia could not be performed because we only included two RCTs (26, 42) of insomnia as measurement outcome. Future studies are warranted to address this issue.

## Conclusion

This comprehensive meta-analysis of 11 RCTs involving a total of 794 participants showed CES is effective in reducing anxiety symptoms with moderate effect size in patients with both primary and secondary anxiety CES was well-tolerated and acceptable.

## Data Availability Statement

The original contributions presented in the study are included in the article/Supplementary Material, further inquiries can be directed to the corresponding author/s.

## Author Contributions

P-YC prepared the manuscript. T-WH and C-SC conceived and designed the study. G-WC and C-CP critically read the manuscript and made important suggestions. P-HC and C-SC took all the responsibility of collecting all the information from the other authors, revised the manuscript, and submitted the manuscript. All authors reviewed the manuscript and had full access to all study data.

## Funding

This work was supported by grants from Kaohsiung Veterans General Hospital, Kaohsiung, Taiwan (KGVGH-110-051, VGHKS-109-070) and the Ministry of Science and Technology, Taiwan (MOST-109-2314-B-075B-001-MY2).

## Conflict of Interest

The authors declare that the research was conducted in the absence of any commercial or financial relationships that could be construed as a potential conflict of interest.

## Publisher's Note

All claims expressed in this article are solely those of the authors and do not necessarily represent those of their affiliated organizations, or those of the publisher, the editors and the reviewers. Any product that may be evaluated in this article, or claim that may be made by its manufacturer, is not guaranteed or endorsed by the publisher.
