# Supplementary material for: Efficacy and Tolerability of Cranial Electrotherapy Stimulation in the Treatment of Anxiety: A Systemic Review and Meta-Analysis
Source: Front Psychiatry. 2022 Jun 9;13:899040. doi: 10.3389/fpsyt.2022.899040 (PMC9218324; doi:10.3389/fpsyt.2022.899040)

**Supplementary Table S1:** Database searches

Date through 2021/07/21

Total: 3025 after removed of duplicated

Pubmed N=134

Keyword: (CES OR Cranial Electrotherapy Stimulation OR cranial electrotherapy stimulation OR noninvasive brain stimulation OR Alpha-Stim) AND (anxiety OR anxious OR panic OR phobia OR worrisome) with limitation on humans species and clinical trial

Medline N= 766

Keyword: (CES OR Cranial Electrotherapy Stimulation OR cranial electrotherapy stimulation OR noninvasive brain stimulation OR Alpha-Stim) AND (anxiety OR anxious OR panic OR phobia OR worrisome)with limitation on humans species

Embase N=1722

Keyword: (CES OR Cranial Electrotherapy Stimulation OR cranial electrotherapy stimulation OR noninvasive brain stimulation OR Alpha-Stim) AND (anxiety OR anxious OR panic OR phobia OR worrisome) ) with limitation on humans and clinical trial

Cochrane library N=403

Keyword: (CES OR Cranial Electrotherapy Stimulation OR cranial electrotherapy stimulation OR noninvasive brain stimulation OR Alpha-Stim) AND (anxiety OR anxious OR panic OR phobia OR worrisome)

**Supplementary Table S2 reasons of excluded trial**

Excluded references with reasons

**Inadequate data: n=31**

1. Morriss R, Price L. Cranial electrotherapy stimulation for the treatment of chronically symptomatic bipolar patients. Journal of Affective Disorders.277:785-8.

2. Morriss R, Xydopoulos G, Craven M, Price L, Fordham R. Clinical effectiveness and cost minimisation model of Alpha-Stim cranial electrotherapy stimulation in treatment seeking patients with moderate to severe generalised anxiety disorder. Journal of Affective Disorders.253:426-37.

3. Roh HT, So WY. Cranial electrotherapy stimulation affects mood state but not levels of peripheral neurotrophic factors or hypothalamic- pituitary-adrenal axis regulation. Technology & Health Care.25(3):403-12.

4. Schmitt R, Capo T, Boyd E. Cranial electrotherapy stimulation as a treatment for anxiety in chemically dependent persons. Alcoholism: Clinical & Experimental Research.10(2):158-60.

5. Smith RB, Tiberi A, Marshall J. The use of cranial electrotherapy stimulation in the treatment of closed-head-injured patients. Brain Injury.8(4):357-61.

6. Winick RL. Cranial electrotherapy stimulation (CES): a safe and effective low cost means of anxiety control in a dental practice. General Dentistry.47(1):50-5.

7. Taylor DN, Lee CT, Katims JJ, Ng LKY. The effects of cranial tens on measures of autonomic, somatic and cognitive activity. Acupuncture and Electro-Therapeutics Research. 1989;14(1):29-42.

8. Taylor DN, Lee CT, Katims JJ. Effects of cranial transcutaneous electrical nerve stimulation in normal subjects at rest and during psychological stress. Acupunct Electrother Res. 1991;16(1-2):65-74.

9. Taylor DN, Lee CT. Frequency-dependent effects of sine-wave cranial transcutaneous electrical nerve stimulation in human subjects. Acupuncture and Electro-Therapeutics Research. 1992;17(3):221-7.

10. Padjen AL, Dongier M, Malec T. Effects of cerebral electrical stimulation on alcoholism: a pilot study. Alcoholism, clinical and experimental research. 1995;19(4):1004‐10.

11. Nct. Cranial Electro Therapy Stimulation in Reducing Perioperative Anxiety. https://clinicaltrialsgov/show/NCT00928772. 2009.

12. Nct. Use of Alpha-Stim Cranial-electrotherapy Stimulation (CES) in the Treatment of Anxiety. https://clinicaltrialsgov/show/NCT01533415. 2012.

13. Amr M, El-Wasify M, Elmaadawi AZ, Roberts RJ, El-Mallakh RS. Cranial electrotherapy stimulation for the treatment of chronically symptomatic bipolar patients. Journal of ECT. 2013;29(2):e31-e2.

14. Tietjen G, Thotakura S, Singh J, Utley C, Ramsey-Williams V, Khubchandani J, et al. Headache prevention with cranial electrotherapy stimulation in chronic migraine. A randomized controlled trial. Cephalalgia. 2013;33(11):963‐4.

15. Irct2015051122176N. Trance cranial direct current electrical stimulation plus cranial electrothrapy stimulation rather than trance cranial direct current stimulation in treatment of major depression disorder. http://wwwwhoint/trialsearch/Trial2aspx?TrialID=IRCT2015051122176N2. 2015.

16. Nct. Combining Non-Invasive Brain Stimulation With Cognitive Behavioral Intervention in Substance Use Disorder. https://clinicaltrialsgov/show/NCT02570763. 2015.

17. Nct. Effectiveness of CES on Emotional and Cellular Wellbeing. https://clinicaltrialsgov/show/NCT03369418. 2016.

18. Nct. The Efficacy of Cranial Electrostimulating Therapy for Depression and Anxiety Among Homeless Adults. https://clinicaltrialsgov/show/NCT02732561. 2016.

19. Jprn U. Exploratory study of optimal conditions of non-invasive brain stimulation for intractable pain. http://wwwwhoint/trialsearch/Trial2aspx?TrialID=JPRN-UMIN000025975. 2017.

20. Nct. Efficacy of CES in New Mothers During the Post Partum Period. https://clinicaltrialsgov/show/NCT03210155. 2017.

21. Nct. A Study of Cranial Electrotherapy Stimulation as an Add-on Treatment for Tic Disorders (SCATT). https://clinicaltrialsgov/show/NCT03705988. 2018.

22. Irct20190307042954N. Comparison of the effects of Chlordiazepoxide and Cranial Electrotherapy Stimulation in preventing the changes of potassium due to surgical anxiety. http://wwwwhoint/trialsearch/Trial2aspx?TrialID=IRCT20190307042954N1. 2019.

23. Kct. Clinical trials evaluating the effect of cranial electrotherapy stimulation (CES) for anxiety and stress reduction. http://wwwwhoint/trialsearch/Trial2aspx?TrialID=KCT0004065. 2019.

24. Nct. Cranial Electrotherapy Stimulation on Anesthetics Consumption and Postoperative Pain. https://clinicaltrialsgov/show/NCT03825471. 2019.

25. Roh D, Kim J, Shin JH. Effects of cranial electrotherapy stimulation with novel in-ear electrodes on anxiety and resting state brain activity: a randomized double-blind placebo-controlled trial. Neuromodulation. 2019;22(7):e401‐.

26. ChiCtr. Effect of Cranial Electrotherapy Stimulation on Postoperative Anxiety of Parturient Undergoing Cesarean Section: a Single-Center, Randomized, Controlled Study. http://wwwwhoint/trialsearch/Trial2aspx?TrialID=ChiCTR2000040963. 2020.

27. Isrctn. Trial investigating the effectiveness of a medical device (Alpha-Stim AID) for the treatment of depressive symptoms for patients in primary care. http://wwwwhoint/trialsearch/Trial2aspx?TrialID=ISRCTN11853110. 2020.

28. Khyatee, Sarkar A, Aggarwal R, Singh I. Impact of cranial electrical stimulation on statistical indices of time domain parameters of heart rate variability in hypertensive individuals. Indian Journal of Public Health Research and Development. 2020;11(3):162-8.

29. Liu EJ, Zhang WL, Wang JB, Zhao FG, Bai YP. Acupuncture combined with cranial electrotherapy stimulation on generalized anxiety disorder: a randomized controlled trial. Zhongguo zhen jiu [Chinese acupuncture & moxibustion]. 2020;40(11):1187‐90.

30. Nct. New Mothers Alpha-Stim. https://clinicaltrialsgov/show/NCT04770181. 2021.

31. Nct. Brain Stimulation & Generalized Anxiety Study. https://clinicaltrialsgov/show/NCT04751864. 2021.

**Study protocol not meet our criteria(n=13)**

1. Kang HW, Kim HJ, Kim WY, Min WK, Min TJ, Lee YS, et al. Effects of cranial electrotherapy stimulation on preoperative anxiety and blood pressure during anesthetic induction in patients with essential hypertension. Journal of International Medical Research.48(8):300060520939370.

2. Lee SH, Kim WY, Lee CH, Min TJ, Lee YS, Kim JH, et al. Effects of cranial electrotherapy stimulation on preoperative anxiety, pain and endocrine response. Journal of International Medical Research.41(6):1788-95.

3. Liu EJ, Zhang WL, Wang JB, Zhao FG, Bai YP. [Acupuncture combined with cranial electrotherapy stimulation on generalized anxiety disorder: a randomized controlled trial]. Zhongguo Zhenjiu.40(11):1187-90.

4. Rogers DR, Ei S, Rogers KR, Cross CL. Evaluation of a multi-component approach to cognitive-behavioral therapy (CBT) using guided visualizations, cranial electrotherapy stimulation, and vibroacoustic sound. Complementary Therapies in Clinical Practice.13(2):95-101.

5. Tegeler CL, Gerdes L, Shaltout HA, Cook JF, Simpson SL, Lee SW, et al. Successful use of closed-loop allostatic neurotechnology for post-traumatic stress symptoms in military personnel: self-reported and autonomic improvements. Military Medical Research.4(1):38.

6. Smith RB, Shiromoto FN. The use of cranial electrotherapy stimulation to block fear perception in phobic patients. Current Therapeutic Research - Clinical and Experimental. 1992;51(2):249-53.

7. Rogers DRB, Ei S, Rogers KR, Cross CL. Evaluation of a multi-component approach to cognitive-behavioral therapy (CBT) using guided visualizations, cranial electrotherapy stimulation, and vibroacoustic sound. Complementary Therapies in Clinical Practice. 2007;13(2):95-101.

8. Kim HJ, Kim WY, Lee YS, Chang MS, Kim JH, Park YC. The effect of cranial electrotherapy stimulation on preoperative anxiety and hemodynamic responses. Korean journal of anesthesiology. 2008;55(6):657‐61.

9. Lee SH, Kim WY, Lee CH, Min TJ, Lee YS, Kim JH, et al. Effects of cranial electrotherapy stimulation on preoperative anxiety, pain and endocrine response. Journal of international medical research. 2013;41(6):1788‐95.

10. Kirsch DL, Price LR, Nichols F, Marksberry JA, Platoni KT. Military service member and veteran self reports of efficacy of cranial electrotherapy stimulation for anxiety, posttraumatic stress disorder, insomnia, and depression. US Army Medical Department journal. 2014:46-54.

11. Ahmari SE. Using mice to model Obsessive Compulsive Disorder: From genes to circuits. Neuroscience. 2016;321:121-37.

12. Efficacy of cranial electrical stimulation and rational emotive behavior therapy in improving psychological illness among chronic stroke survivors: a pilot randomized controlled trial. Annals of indian academy of neurology. 2018;21(3):188‐92.

13. Kang HW, Kim HJ, Kim WY, Min WK, Min TJ, Lee YS, et al. Effects of cranial electrotherapy stimulation on preoperative anxiety and blood pressure during anesthetic induction in patients with essential hypertension. Journal of international medical research. 2020;48(8):300060520939370.

**Other intervention(n = 327)**

1. Ahn H, Sorkpor S, Miao H, Zhong C, Jorge R, Park L, et al. Home-based self-administered transcranial direct current stimulation in older adults with knee osteoarthritis pain: An open-label study. Journal of Clinical Neuroscience.66:61-5.

2. Bennebroek Evertsz F, Bockting CL, Stokkers PC, Hinnen C, Sanderman R, Sprangers MA. The effectiveness of cognitive behavioral therapy on the quality of life of patients with inflammatory bowel disease: multi-center design and study protocol (KL!C- study). BMC Psychiatry.12:227.

3. Boggio PS, Rocha M, Oliveira MO, Fecteau S, Cohen RB, Campanha C, et al. Noninvasive brain stimulation with high-frequency and low-intensity repetitive transcranial magnetic stimulation treatment for posttraumatic stress disorder. Journal of Clinical Psychiatry.71(8):992-9.

4. Bohlmeijer ET, Fledderus M, Rokx TA, Pieterse ME. Efficacy of an early intervention based on acceptance and commitment therapy for adults with depressive symptomatology: Evaluation in a randomized controlled trial. Behaviour Research & Therapy.49(1):62-7.

5. Bolier L, Haverman M, Kramer J, Westerhof GJ, Riper H, Walburg JA, et al. An Internet-based intervention to promote mental fitness for mildly depressed adults: randomized controlled trial. Journal of Medical Internet Research.15(9):e200.

6. Bolinski F, Kleiboer A, Karyotaki E, Bosmans JE, Zarski AC, Weisel KK, et al. Effectiveness of a transdiagnostic individually tailored Internet-based and mobile-supported intervention for the indicated prevention of depression and anxiety (ICare Prevent) in Dutch college students: study protocol for a randomised controlled trial. Trials [Electronic Resource].19(1):118.

7. Borza L. Cognitive-behavioral therapy for generalized anxiety. Dialogues in Clinical Neuroscience.19(2):203-8.

8. Brandao Filho RA, Baptista AF, Brandao Rde A, Meneses FM, Okeson J, de Sena EP. Analgesic effect of cathodal transcranial current stimulation over right dorsolateral prefrontal cortex in subjects with muscular temporomandibular disorders: study protocol for a randomized controlled trial. Trials [Electronic Resource].16:415.

9. Carnevali L, Pattini E, Sgoifo A, Ottaviani C. Effects of prefrontal transcranial direct current stimulation on autonomic and neuroendocrine responses to psychosocial stress in healthy humans. Stress.23(1):26-36.

10. Christensen H, Batterham P, Mackinnon A, Griffiths KM, Kalia Hehir K, Kenardy J, et al. Prevention of generalized anxiety disorder using a web intervention, iChill: randomized controlled trial. Journal of Medical Internet Research.16(9):e199.

11. Chukhlovina ML, Chukhlovin AA. [Assessment of clinical manifestations and treatment of post-stroke depression in young patients with ischemic stroke]. Zhurnal Nevrologii i Psikhiatrii Imeni SS Korsakova.118(3):52-5.

12. Cooney LG, Milman LW, Hantsoo L, Kornfield S, Sammel MD, Allison KC, et al. Cognitive-behavioral therapy improves weight loss and quality of life in women with polycystic ovary syndrome: a pilot randomized clinical trial. Fertility & Sterility.110(1):161-71.e1.

13. de Lima AL, Braga FMA, da Costa RMM, Gomes EP, Brunoni AR, Pegado R. Transcranial direct current stimulation for the treatment of generalized anxiety disorder: A randomized clinical trial. Journal of Affective Disorders.259:31-7.

14. Dozeman E, van Schaik DJ, van Marwijk HW, Stek ML, Beekman AT, van der Horst HE. Feasibility and effectiveness of activity-scheduling as a guided self-help intervention for the prevention of depression and anxiety in residents in homes for the elderly: a pragmatic randomized controlled trial. International Psychogeriatrics.23(6):969-78.

15. Fountoulakis KN, Karavelas V, Moysidou S, Mavridis D, Pastiadis K, Petalidou N, et al. Efficacy of Add-on Pregabalin in the Treatment of Patients with Generalized Anxiety Disorder and Unipolar Major Depression With an Early Nonresponse to Escitalopram: A Double-Blind Placebo-Controlled Study. Pharmacopsychiatry.52(4):193-202.

16. Guo T, Guo Z, Zhang W, Ma W, Yang X, Yang X, et al. Electroacupuncture and cognitive behavioural therapy for sub-syndromal depression among undergraduates: a controlled clinical trial. Acupuncture in Medicine.34(5):356-63.

17. Jacquemin L, Shekhawat GS, Van de Heyning P, Mertens G, Fransen E, Van Rompaey V, et al. Effects of Electrical Stimulation in Tinnitus Patients: Conventional Versus High-Definition tDCS. Neurorehabilitation & Neural Repair.32(8):714-23.

18. Joling KJ, van Hout HP, Scheltens P, Vernooij-Dassen M, van den Berg B, Bosmans J, et al. (Cost)-effectiveness of family meetings on indicated prevention of anxiety and depressive symptoms and disorders of primary family caregivers of patients with dementia: design of a randomized controlled trial. BMC Geriatrics.8:2.

19. Kaczkurkin AN, Foa EB. Cognitive-behavioral therapy for anxiety disorders: an update on the empirical evidence. Dialogues in Clinical Neuroscience.17(3):337-46.

20. Kerper LF, Spies CD, Losner M, Salz AL, Tafelski S, Balzer F, et al. Persistence of psychological distress in surgical patients with interest in psychotherapy: results of a 6-month follow-up. PLoS ONE [Electronic Resource].7(12):e51167.

21. Kleiboer A, Donker T, Seekles W, van Straten A, Riper H, Cuijpers P. A randomized controlled trial on the role of support in Internet-based problem solving therapy for depression and anxiety. Behaviour Research & Therapy.72:63-71.

22. Kok RN, van Straten A, Beekman AT, Cuijpers P. Short-term effectiveness of web-based guided self-help for phobic outpatients: randomized controlled trial. Journal of Medical Internet Research.16(9):e226.

23. Lefaucheur JP, Andre-Obadia N, Antal A, Ayache SS, Baeken C, Benninger DH, et al. Evidence-based guidelines on the therapeutic use of repetitive transcranial magnetic stimulation (rTMS). Clinical Neurophysiology.125(11):2150-206.

24. Loi CXA, Nesman TM, Xu P, Taylor TR, McMillan S, Krischer JP, et al. A Self-Administered Stress Management Intervention for Hispanic Patients Undergoing Cancer Chemotherapy. Journal of Immigrant & Minority Health.19(5):1121-31.

25. Macaulay C, Angus L, Khattra J, Westra H, Ip J. Client Retrospective Accounts of Corrective Experiences in Motivational Interviewing Integrated With Cognitive Behavioral Therapy for Generalized Anxiety Disorder. Journal of Clinical Psychology.73(2):168-81.

26. Macedo IC, de Oliveira C, Vercelino R, Souza A, Laste G, Medeiros LF, et al. Repeated transcranial direct current stimulation reduces food craving in Wistar rats. Appetite.103:29-37.

27. Malavera A, Silva FA, Fregni F, Carrillo S, Garcia RG. Repetitive Transcranial Magnetic Stimulation for Phantom Limb Pain in Land Mine Victims: A Double-Blinded, Randomized, Sham-Controlled Trial. Journal of Pain.17(8):911-8.

28. Mannu P, Rinaldi S, Fontani V, Castagna A, Margotti ML. Radio electric treatment vs. Es-Citalopram in the treatment of panic disorders associated with major depression: an open-label, naturalistic study. Acupuncture & Electro-Therapeutics Research.34(3-4):135-49.

29. Nagano M, Shimizu K, Kondo R, Hayashi C, Sato D, Kitagawa K, et al. Reduction of depression and anxiety by 4 weeks Hericium erinaceus intake. Biomedical Research.31(4):231-7.

30. Noguchi R, Sekizawa Y, So M, Yamaguchi S, Shimizu E. Effects of five-minute internet-based cognitive behavioral therapy and simplified emotion-focused mindfulness on depressive symptoms: a randomized controlled trial. BMC Psychiatry.17(1):85.

31. Palm U, Leitner B, Kirsch B, Behler N, Kumpf U, Wulf L, et al. Prefrontal tDCS and sertraline in obsessive compulsive disorder: a case report and review of the literature. Neurocase.23(2):173-7.

32. Park JS, Park S, Cheon CH, Jang BH, Lee SH, Lee SH, et al. Effect of oriental medicine music therapy on patients with Hwa-byung: a study protocol for a randomized controlled trial. Trials [Electronic Resource].13:161.

33. Philip NS, Leuchter AF, Cook IA, Massaro J, Goethe JW, Carpenter LL. Predictors of response to synchronized transcranial magnetic stimulation for major depressive disorder. Depression & Anxiety.36(3):278-85.

34. Prytys M, Harman K, Lee R, Brown JS. Who attends and who benefits from CBT "self-confidence" workshops run in routine practice? A pilot study. Behavioural & Cognitive Psychotherapy.37(5):585-93.

35. Roepke AM, Jaffee SR, Riffle OM, McGonigal J, Broome R, Maxwell B. Randomized Controlled Trial of SuperBetter, a Smartphone-Based/Internet-Based Self-Help Tool to Reduce Depressive Symptoms. Games for Health Journal.4(3):235-46.

36. Rzheusskaia GV, Listopadov Iu I, Bobrova MV, Umrudina AG. [Cymbalta (duloxetine) in the treatment of anxiety-depressive disorders in patients with discirculatory encephalopathy]. Zhurnal Nevrologii i Psikhiatrii Imeni SS Korsakova.109(2):26-30.

37. Sarkar A, Dowker A, Cohen Kadosh R. Cognitive enhancement or cognitive cost: trait-specific outcomes of brain stimulation in the case of mathematics anxiety. Journal of Neuroscience.34(50):16605-10.

38. Smits FM, de Kort GJ, Geuze E. Acceptability of tDCS in treating stress-related mental health disorders: a mixed methods study among military patients and caregivers. BMC Psychiatry.21(1):97.

39. van der Zanden RA, Kramer JJ, Cuijpers P. Effectiveness of an online group course for adolescents and young adults with depressive symptoms: study protocol for a randomized controlled trial. Trials [Electronic Resource].12:196.

40. van Straten A, Cuijpers P, Smits N. Effectiveness of a web-based self-help intervention for symptoms of depression, anxiety, and stress: randomized controlled trial. Journal of Medical Internet Research.10(1):e7.

41. van 't Veer-Tazelaar N, van Marwijk H, van Oppen P, Nijpels G, van Hout H, Cuijpers P, et al. Prevention of anxiety and depression in the age group of 75 years and over: a randomised controlled trial testing the feasibility and effectiveness of a generic stepped care programme among elderly community residents at high risk of developing anxiety and depression versus usual care [ISRCTN26474556]. BMC Public Health.6:186.

42. Van't Wout M, Longo SM, Reddy MK, Philip NS, Bowker MT, Greenberg BD. Transcranial direct current stimulation may modulate extinction memory in posttraumatic stress disorder. Brain and Behavior.7(5):e00681.

43. Weisel KK, Zarski AC, Berger T, Krieger T, Moser CT, Schaub MP, et al. User Experience and Effects of an Individually Tailored Transdiagnostic Internet-Based and Mobile-Supported Intervention for Anxiety Disorders: Mixed-Methods Study. Journal of Medical Internet Research.22(9):e16450.

44. Welch MG, Halperin MS, Austin J, Stark RI, Hofer MA, Hane AA, et al. Depression and anxiety symptoms of mothers of preterm infants are decreased at 4 months corrected age with Family Nurture Intervention in the NICU. Archives of Women's Mental Health.19(1):51-61.

45. Zorzo C, Banqueri M, Higarza SG, Pernia AM, Arias JL. Current State of Transcranial Magnetic Stimulation and its use in Psychiatry. Actas Espanolas de Psiquiatria.47(3):110-20.

46. George MS, Nahas Z, Molloy M, Speer AM, Oliver NC, Li XB, et al. A controlled trial of daily left prefrontal cortex TMS for treating depression. Biological psychiatry. 2000;48(10):962‐70.

47. Lisanby SH, Kinnunen LH, Crupain MJ. Applications of TMS to therapy in psychiatry. Journal of Clinical Neurophysiology. 2002;19(4):344-60.

48. George MS, Nahas Z, Lisanby SH, Schlaepfer T, Kozel FA, Greenberg BD. Transcranial magnetic stimulation. Neurosurgery Clinics of North America. 2003;14(2):283-301.

49. Fregni F, Boggio PS, Lima MC, Ferreira MJ, Wagner T, Rigonatti SP, et al. A sham-controlled, phase II trial of transcranial direct current stimulation for the treatment of central pain in traumatic spinal cord injury. Pain. 2006;122(1‐2):197‐209.

50. Fregni F, Boggio PS, Nitsche MA, Rigonatti SP, Pascual-Leone A. Cognitive effects of repeated sessions of transcranial direct current stimulation in patients with depression [1]. Depression and Anxiety. 2006;23(8):482-4.

51. Fregni F, Gimenes R, Valle AC, Ferreira MJ, Rocha RR, Natalle L, et al. A randomized, sham-controlled, proof of principle study of transcranial direct current stimulation for the treatment of pain in fibromyalgia. Arthritis Rheum. 2006;54(12):3988-98.

52. Haringsma R, Engels GI, Cuijpers P, Spinhoven P. Effectiveness of the Coping With Depression (CWD) course for older adults provided by the community-based mental health care system in the Netherlands: a randomized controlled field trial. International psychogeriatrics. 2006;18(2):307‐25.

53. Nct. Transcranial Magnetic Stimulation (TMS) Effects on Pain Perception. https://clinicaltrialsgov/show/NCT00349050. 2006.

54. Camprodon JA, Martínez-Raga J, Alonso-Alonso M, Shih MC, Pascual-Leone A. One session of high frequency repetitive transcranial magnetic stimulation (rTMS) to the right prefrontal cortex transiently reduces cocaine craving. Drug and alcohol dependence. 2007;86(1):91‐4.

55. Magierski R, Kłoszewska I. Applications of transcranial magnetic stimulation in psychiatry. Aktualnosci Neurologiczne. 2007;7(1):32-9.

56. Nct. Repetitive Transcranial Magnetic Stimulation (rTMS) in the Treatment of Panic Disorder With Comorbid Major Depression. https://clinicaltrialsgov/show/NCT00521352. 2007.

57. Passard A, Attal N, Benadhira R, Brasseur L, Saba G, Sichere P, et al. Effects of unilateral repetitive transcranial magnetic stimulation of the motor cortex on chronic widespread pain in fibromyalgia. Brain. 2007;130(Pt 10):2661‐70.

58. Bystritsky A, Kaplan JT, Feusner JD, Kerwin LE, Wadekar M, Burock M, et al. A preliminary study of fMRI-guided rTMS in the treatment of generalized anxiety disorder. Journal of Clinical Psychiatry. 2008;69(7):1092-8.

59. Joling KJ, van Hout HP, Scheltens P, Vernooij-Dassen M, van den Berg B, Bosmans J, et al. (Cost)-effectiveness of family meetings on indicated prevention of anxiety and depressive symptoms and disorders of primary family caregivers of patients with dementia: design of a randomized controlled trial. BMC geriatrics. 2008;8:2.

60. Nct. The Use of rTMS to Improve Theory of Mind Among Adults With Autism and Asperger's Disorder. https://clinicaltrialsgov/show/NCT00808782. 2008.

61. Pigot M, Loo C, Sachdev P. Repetitive transcranial magnetic stimulation as treatment for anxiety disorders. Expert Review of Neurotherapeutics. 2008;8(10):1449-55.

62. Shah DB, Weaver L, O'Reardon JP. Transcranial magnetic stimulation: A device intended for the psychiatrist's office, but what is its future clinical role? Expert Review of Medical Devices. 2008;5(5):559-66.

63. Boggio PS, Zaghi S, Fregni F. Modulation of emotions associated with images of human pain using anodal transcranial direct current stimulation (tDCS). Neuropsychologia. 2009;47(1):212-7.

64. Chae J, Lee SJ, Seo HJ, Jeong YE. Efficacy and safety of repeated TMS in the treatment of refractory obsessive-compulsive disorder. European Neuropsychopharmacology. 2009;19:S593.

65. Cohen RB, Boggio PS, Fregni F. Risk factors for relapse after remission with repetitive transcranial magnetic stimulation for the treatment of depression. Depression and Anxiety. 2009;26(7):682-8.

66. Lim MH, Kim HW, Lee KK, Paik KC, Lee SB, Chae JH. Repeated transcranial magnetic stimulation in Tourette's syndrome. European Neuropsychopharmacology. 2009;19:S680-S1.

67. Mannu P, Rinaldi S, Fontani V, Castagna A, Margotti ML. Radio electric treatment vs. Es-Citalopram in the treatment of panic disorders associated with major depression: an open-label, naturalistic study. Acupuncture & electro-therapeutics research. 2009;34(3‐4):135‐49.

68. Ntr. Enhancing the quality of life of patients with inflammatory bowel disease: a multi-center study investigating cognitive behavioral therapy. http://wwwwhoint/trialsearch/Trial2aspx?TrialID=NTR1869. 2009.

69. Prytys M, Harman K, Lee R, Brown JS. Who attends and who benefits from CBT "self-confidence" workshops run in routine practice? A pilot study. Behavioural and cognitive psychotherapy. 2009;37(5):585-93.

70. Rzheusskaya GV, Listopadov YUI, Bobrova MV, Umrudina AG. Cymbalta (duloxetine) in the treatment of anxiety-depressive disorders in patients with discirculatory encephalopathy. Zhurnal Nevrologii i Psihiatrii imeni SS Korsakova. 2009;109(2):26-30.

71. Soekadar SR, Arfeller C, Rilk A, Plontke SK, Plewnia C. Theta burst stimulation in the treatment of incapacitating tinnitus accompanied by severe depression. CNS Spectrums. 2009;14(4):208-11.

72. Zanoni S, Castellano F, D'Urso N, Dobrea C, Benatti B, Arici C, et al. Transcranial direct current stimulation (tDCS) in patients with Major Depressive Episode. International Journal of Psychiatry in Clinical Practice. 2009;13(SUPPL. 1):47.

73. Boggio PS, Rocha M, Oliveira MO, Fecteau S, Cohen RB, Campanhã C, et al. Noninvasive brain stimulation with high-frequency and low-intensity repetitive transcranial magnetic stimulation treatment for posttraumatic stress disorder. Journal of clinical psychiatry. 2010;71(8):992‐9.

74. Chi CT. A randomized, controlled clinical trail: the effects of mindfulness-based cognitive therapy on generalized anxiety disorder and health service utilization in primary care. http://wwwwhoint/trialsearch/Trial2aspx?TrialID=ChiCTR-TRC-10000907. 2010.

75. Choi S, Kim DY, Whang SH, Lee JH, Hann SK, Shin YJ. Quality of life and psychological adaptation of Korean adolescents with vitiligo: ORIGINAL ARTICLE. Journal of the European Academy of Dermatology and Venereology. 2010;24(5):524-9.

76. Mori F, Codecà C, Kusayanagi H, Monteleone F, Buttari F, Fiore S, et al. Effects of anodal transcranial direct current stimulation on chronic neuropathic pain in patients with multiple sclerosis. Journal of pain. 2010;11(5):436‐42.

77. Soler D, Kumru H, Vidal J, Fregni F, Tormos JM, Navarro X, et al. Transcranial direct current stimulation (TDCS) and virtual reality (VR) techniques for treatment neuropathic central pain in spinal cord injury (NP-SCI). European Journal of Pain Supplements. 2010;4(1):105-6.

78. Triggs WJ, Ricciuti N, Ward HE, Cheng J, Bowers D, Goodman WK, et al. Right and left dorsolateral pre-frontal rTMS treatment of refractory depression: A randomized, sham-controlled trial. Psychiatry Research. 2010;178(3):467-74.

79. Arias-Carrión O, Machado S, Paes F, Velasques B, Teixeira S, Cárdenas-Morales L, et al. Is rTMS an effective therapeutic strategy that can be used to treat Parkinson's disease? CNS and Neurological Disorders - Drug Targets. 2011;10(6):693-702.

80. Dozeman E, van Schaik DJ, van Marwijk HW, Stek ML, Beekman AT, van der Horst HE. Feasibility and effectiveness of activity-scheduling as a guided self-help intervention for the prevention of depression and anxiety in residents in homes for the elderly: a pragmatic randomized controlled trial. International psychogeriatrics. 2011;23(6):969‐78.

81. Ferrucci R, Bortolomasi M, Fumagalli M, Giacopuzzi M, Mameli F, Priori A, et al. Transcranial direct current stimulation (tDCS) - A treatment for depression. International Journal of Psychiatry in Clinical Practice. 2011;15:23.

82. Fontani V, Mannu P, Castagna A, Rinaldi S. Social anxiety disorder: Radio electric asymmetric conveyor brain stimulation versus sertraline. Patient Preference and Adherence. 2011;5:581-6.

83. Luedtke K, Rushton A, Wright C, Juergens TP, Mueller G, May A. Effectiveness of anodal transcranial direct current stimulation in patients with chronic low back pain: design, method and protocol for a randomised controlled trial. BMC Musculoskelet Disord. 2011;12:290.

84. Mannu P, Rinaldi S, Fontani V, Castagna A, Margotti ML. Noninvasive brain stimulation by radioelectric asymmetric conveyor in the treatment of agoraphobia: Open-label, naturalistic study. Patient Preference and Adherence. 2011;5:575-80.

85. Mishra BR, Sarkar S, Praharaj SK, Mehta VS, Diwedi S, Haque Nizamie S. Repetitive transcranial magnetic stimulation in psychiatry. Annals of Indian Academy of Neurology. 2011;14(4):245-51.

86. Nct. Synchronized Transcranial Magnetic Stimulation (sTMS) in Major Depressive Disorder. https://clinicaltrialsgov/show/NCT01370733. 2011.

87. Notaristefano A, Henin M, Bottero G, Politi E, Smeraldi E. Effects of transcranial neuromagnetic and neuroelectical stimulation on alcohol craving. European Neuropsychopharmacology. 2011;21:S565-S6.

88. Sampson SM, Kung S, McAlpine DE, Sandroni P. The use of slow-frequency prefrontal repetitive transcranial magnetic stimulation in refractory neuropathic pain. Journal of ECT. 2011;27(1):33-7.

89. Tadini L, El-Nazer R, Brunoni AR, Williams J, Carvas M, Boggio P, et al. Cognitive, mood, and electroencephalographic effects of noninvasive cortical stimulation with weak electrical currents. Journal of ECT. 2011;27(2):134‐40.

90. Adeyemo BO. Assessment of a revolutionary safety comparative model of multiple agents of non-invasive stimulation. PM and R. 2012;4(10):S373.

91. Durmaz O, Ateş MA, Çetin M, Başoʇlu C, Ebrinc¸ S, Algül A. Is rTMS more effective in treating comorbid anxiety and somatic symptoms of unipolar refractory major depression? Klinik Psikofarmakoloji Bulteni. 2012;22:S36.

92. Jprn U. A sham-controlled, double-blind, parallel-group, randomized trial of repetitive transcranial magnetic stimulation (rTMS) for major depressive disorder, followed by a semi-crossover open trial of rTMS. http://wwwwhoint/trialsearch/Trial2aspx?TrialID=JPRN-UMIN000007794. 2012.

93. MacHado S, Paes F, Velasques B, Teixeira S, Piedade R, Ribeiro P, et al. Is rTMS an effective therapeutic strategy that can be used to treat anxiety disorders? Neuropharmacology. 2012;62(1):125-34.

94. Berger C, Domning T, Thome J, Höppner J. rTMS and eeg-investigations in healthy subjects. Clinical Neurophysiology. 2013;124(10):e76-e7.

95. Brunoni AR, Vanderhasselt MA, Boggio PS, Fregni F, Dantas EM, Mill JG, et al. Polarity- and valence-dependent effects of prefrontal transcranial direct current stimulation on heart rate variability and salivary cortisol. Psychoneuroendocrinology. 2013;38(1):58-66.

96. da Silva MC, Conti CL, Klauss J, Alves LG, do Nascimento Cavalcante HM, Fregni F, et al. Behavioral effects of transcranial Direct Current Stimulation (tDCS) induced dorsolateral prefrontal cortex plasticity in alcohol dependence. Journal of Physiology Paris. 2013;107(6):493-502.

97. D'Urso G, Dell'Osso B, Ferrucci R, Bortolomasi M, Bruzzese D, Giacopuzzi M, et al. Transcranial direct current stimulation (tDCS) for the treatment of major depression: A pooled analysis from the Italian tDCS collaborative group. Clinical Neurophysiology. 2013;124(10):e185.

98. Eryilmaz G, Onen Unsalver B, Gogcegoz G, Saglam E. Demographic evaluation of patients treated with transcranial direct current stimulation (TDCS) in a university hospital. European neuropsychopharmacology. 2013;23:S242‐S3.

99. Eryilmaz G, Sayar GH, Unsalver BO, Gul IG, Ozten E, Saglam E. A retrospective review assessing the safety of transcranial direct current stimulation (tDCS) as a method of non-invasive brain stimulation in psychiatry. Klinik Psikofarmakoloji Bulteni. 2013;23:S140-S1.

100. Field T, Diego M, Delgado J, Medina L. Tai chi/yoga reduces prenatal depression, anxiety and sleep disturbances. Complementary therapies in clinical practice. 2013;19(1):6‐10.

101. Field T, Diego M, Delgado J, Medina L. Peer support and interpersonal psychotherapy groups experienced decreased prenatal depression, anxiety and cortisol. Early human development. 2013;89(9):621‐4.

102. Li CT, Su TP, Hsieh JC, Ho ST. Efficacy and practical issues of repetitive transcranial magnetic stimulation on chronic medically unexplained symptoms of pain. Acta Anaesthesiologica Taiwanica. 2013;51(2):81-7.

103. Luedtke K, Rushton A, Wright C, Jürgens T, Mueller G, May A. Effectiveness of anodal transcranial direct current stimulation in patients with chronic low back pain: Design, method and protocol for a randomised controlled trial. Clinical Neurophysiology. 2013;124(10):e118.

104. Mantovani A, Shubeck J, Gowatsky J, Simpson HB, Greenberg B. Incompleteness and harm avoidance in obsessive compulsive disorder: Different response to transcranial magnetic stimulation. Clinical Neurophysiology. 2013;124(10):e182-e3.

105. Mylius V, Ayache SS, Farhat WH, Zouari HG, Passeri E, Aoun-Sebaïti M, et al. Robotized-navigated low-frequency repetitive transcranial magnetic stimulation over the right motor and prefrontal cortex improved pain and fatigue in patients with macrophagic myofasciitis. Clinical Neurophysiology. 2013;124(10):e116.

106. Nct. tDCS Applied to the OFC: effects on Decision-Making and Impulse Control. https://clinicaltrialsgov/show/NCT01805401. 2013.

107. Stern AP, Cohen D. Repetitive transcranial magnetic stimulation for treatment-resistant depression. Neuropsychiatry. 2013;3(1):107-15.

108. Villamar MF, Wivatvongvana P, Patumanond J, Bikson M, Truong DQ, Datta A, et al. Focal modulation of the primary motor cortex in fibromyalgia using 4×1-ring high-definition transcranial direct current stimulation (HD-tDCS): Immediate and delayed analgesic effects of cathodal and anodal stimulation. Journal of Pain. 2013;14(4):371-83.

109. Amos T, Stein DJ, Ipser JC. Pharmacological interventions for preventing post‐traumatic stress disorder (PTSD). Cochrane Database of Systematic Reviews. 2014(7).

110. Anderkova L, Rektorova I. Cognitive effects of repetitive transcranial magnetic stimulation in patients with neurodegenerative diseases - Clinician's perspective. Journal of the Neurological Sciences. 2014;339(1-2):15-25.

111. Bennabi D, Pedron S, Haffen E, Monnin J, Peterschmitt Y, Van Waes V. Transcranial direct current stimulation for memory enhancement: From clinical research to animal models. Frontiers in Systems Neuroscience. 2014;8(SEP).

112. Camprodon J, Kaur N, Deckersbach T, Evans K, Kopell B, Halverson J, et al. Epidural cortical stimulation of the left DLPFC leads to dose-dependent enhancement of working memory in patients with MDD. Neuropsychopharmacology. 2014;39:S565.

113. Chae JH. Repetitive transcranial magnetic stimulation treatment studies in depression and anxiety disorders. Bipolar Disorders. 2014;16:59-60.

114. Dalla Libera D, Petrillo C, Albini A, Rossi P, Ruggieri G. Transcranic magnetic stimulation enhances the potential of rehabilitative multidisciplinary treatment in chronic pain patients. Clinical Neurophysiology. 2014;125:S317-S8.

115. Jprn U. A semi-crossover open trial of combined therapy of fMRI neurofeedback and repetitive transcranial magnetic stimulation (rTMS) for affective disorders. http://wwwwhoint/trialsearch/Trial2aspx?TrialID=JPRN-UMIN000013272. 2014.

116. Jprn U. Effects of a 5minutes internet-based cognitive behavioral therapy (iCBT) and easy mindfulness exercise intervention on depressive mood : a large scale randomized controlled trial. http://wwwwhoint/trialsearch/Trial2aspx?TrialID=JPRN-UMIN000015097. 2014.

117. Klauss J, Penido Pinheiro LC, Silva Merlo BL, Correia Santos GDA, Fregni F, Nitsche MA, et al. A randomized controlled trial of targeted prefrontal cortex modulation with tDCS in patients with alcohol dependence. International Journal of Neuropsychopharmacology. 2014;17(11):1793-803.

118. Kok RN, van Straten A, Beekman AT, Cuijpers P. Short-term effectiveness of web-based guided self-help for phobic outpatients: randomized controlled trial. Journal of medical Internet research. 2014;16(9):e226.

119. Kuo MF, Paulus W, Nitsche MA. Therapeutic effects of non-invasive brain stimulation with direct currents (tDCS) in neuropsychiatric diseases. NeuroImage. 2014;85:948-60.

120. Lefaucheur JP, André-Obadia N, Antal A, Ayache SS, Baeken C, Benninger DH, et al. Evidence-based guidelines on the therapeutic use of repetitive transcranial magnetic stimulation (rTMS). Clinical Neurophysiology. 2014;125(11):2150-206.

121. Losada A, Marquez-Gonzalez M, Romero-Moreno R, Lopez J, Fernandez-Fernandez V, Nogales-Gonzalez C. Cognitive behavioral therapy (CBT) versus acceptance and commitment therapy (ACT) for dementia family caregivers: follow-up results of a randomized clinical trial. European geriatric medicine. 2014;5:S46.

122. Ma XY, Huang YQ, Liao LW, Jin Y. A randomized double-blinded sham-controlled trial of α electroencephalogram-guided transcranial magnetic stimulation for obsessive-compulsive disorder. Chinese Medical Journal. 2014;127(4):601-6.

123. Miler JA, Meron D, Garner M, Baldwin DS. The effect of prefrontal transcranial direct current stimulation (tDCS) on attention network function in healthy humans. European Neuropsychopharmacology. 2014;24:S82.

124. Nct. Analgesic Effect of Cathodal tDCS Over Right DLPFC in Subjects With Muscular TMD: a Double Blind Crossover RCT. https://clinicaltrialsgov/show/NCT02152267. 2014.

125. Zwanzger P, Steinberg C, Rehbein MA, Bröckelmann AK, Dobel C, Zavorotnyy M, et al. Inhibitory repetitive transcranial magnetic stimulation (rTMS) of the dorsolateral prefrontal cortex modulates early affective processing. NeuroImage. 2014;101:193-203.

126. Batista EK, Klauss J, Fregni F, Nitsche MA, Nakamura-Palacios EM. A randomized placebo-controlled trial of targeted prefrontal cortex modulation with bilateral tDCS in patients with crack-cocaine dependence. International Journal of Neuropsychopharmacology. 2015;18(12):1-11.

127. Brandão Filho RA, Baptista AF, Brandão RAFS, Meneses FM, Okeson J, de Sena EP. Analgesic effect of cathodal transcranial current stimulation over right dorsolateral prefrontal cortex in subjects with muscular temporomandibular disorders: Study protocol for a randomized controlled trial. Trials. 2015;16(1).

128. Camprodon JA, Kaur N, Deckersbach T, Evans KC, Kopell BH, Halverson J, et al. Epidural cortical stimulation of the left DLPFC leads to dose-dependent enhancement of working memory in patients with MDD. Brain Stimulation. 2015;8(2):408.

129. Chung SW, Hoy KE, Fitzgerald PB. Theta-burst stimulation: A new form of tms treatment for depression? Depression and Anxiety. 2015;32(3):182-92.

130. Clark C, Cole J, Winter C, Williams K, Grammer G. A Review of Transcranial Magnetic Stimulation as a Treatment for Post-Traumatic Stress Disorder. Current Psychiatry Reports. 2015;17(10).

131. Dastjerdi G, Mirhoseini H, Mohammadi E. Investigating the synergistic effects of transcranial direct current stimulation and cranial electrical stimulation in treatment of major depression in a double blinded controlled trial. Biomedical and pharmacology journal. 2015;8(2):1267‐74.

132. Dauchy S, Majed L, Saltel P, Reich M, Rouby P, Lopez C, et al. A randomized, double-blind, placebo-controlled trial of escitalopram for the treatment of emotional distress during treatment for head and neck cancer. Psycho-Oncology. 2015;24:332.

133. Lin YC, Feng Y, Zhan SQ, Li N, Ding Y, Hou Y, et al. Repetitive transcranial magnetic stimulation for the treatment of restless legs syndrome. Chinese Medical Journal. 2015;128(13):1728-31.

134. McClelland J, Campbell IC, Schmidt U. A randomised, double blind controlled trial of neuronavigated repetitive transcranial magnetic stimulation in anorexia nervosa. European Neuropsychopharmacology. 2015;25:S64-S5.

135. McKendrick R, Parasuraman R, Ayaz H. Wearable functional near infrared spectroscopy (fNIRS) and transcranial direct current stimulation (tDCS): Expanding vistas for neurocognitive augmentation. Frontiers in Systems Neuroscience. 2015;9(MAR).

136. Mukherjee N, Sinha VK, Goyal N. Efficacy of adjunctive neuronavigated repetitive trans cranial magnetic stimulation(rTMS) in localization related epilepsy in children and adolescents: A sham-controlled study. Indian Journal of Psychiatry. 2015;57(5):S30.

137. Nct. Transcranial Direct Current Stimulation Apparatus for Domiciliary Use. https://clinicaltrialsgov/show/NCT02408237. 2015.

138. Nct. Home-based Transcranial Direct Current Stimulation in Fibromyalgia Patients. https://clinicaltrialsgov/show/NCT02652988. 2015.

139. Nct. Cerebral Hemodynamics With rTMS in Alcohol Dependence. https://clinicaltrialsgov/show/NCT02446067. 2015.

140. Nizard J, Levesque A, Denis N, De Chauvigny E, Lepeintre A, Raoul S, et al. Interest of repetitive transcranial magnetic stimulation of the motor cortex in the management of refractory cancer pain in palliative care: Two case reports. Palliative Medicine. 2015;29(6):564-8.

141. O'Neill F, Sacco P, Nurmikko T. Evaluation of a home-based transcranial direct current stimulation (tDCS) treatment device for chronic pain: study protocol for a randomised controlled trial. Trials. 2015;16:186.

142. Palm U, Strube W, Bunse T, Bauer I, Dunkel G, Hasan A, et al. Transcranial direct current stimulation in psychiatry - Update 2015. Nervenheilkunde. 2015;34(12):1016-25.

143. Pradhan S, Kirton A, MacQueen G, MacMaster F. The effect of repetitive transcranial magnetic stimulation on dorsolateral prefrontal glutamate in youth with treatment-resistant depression. BMC Proceedings. 2015;9.

144. Van 't Wout M, Mariano TY, Reddy MK, Rasmussen SA, Greenberg B. Modulating extinction of conditioned fear by transcranial direct current stimulation. Biological Psychiatry. 2015;77(9):43S-4S.

145. Blumberger DM, Maller JJ, Thomson L, Mulsant BH, Rajji TK, Maher M, et al. Unilateral and bilateral MRI-targeted repetitive transcranial magnetic stimulation for treatmentresistant depression: A randomized controlled study. Journal of Psychiatry and Neuroscience. 2016;41(4):E58-E66.

146. Caulfield KA, Ketchabaw WT, Pascual-Leone A, Press DZ, Stern AP. Reductions in depression and anxiety measures are correlated in patients receiving transcranial magnetic stimulation. Brain Stimulation. 2016;9(5):e9.

147. Cha YH, Urbano D, Pariseau N. Randomized Single Blind Sham Controlled Trial of Adjunctive Home-Based tDCS after rTMS for Mal De Debarquement Syndrome: Safety, Efficacy, and Participant Satisfaction Assessment. Brain Stimulation. 2016;9(4):537-44.

148. Chabardès S, Carron R, Seigneuret E, Torres N, Goetz L, Krainik A, et al. Endoventricular Deep Brain Stimulation of the Third Ventricle: Proof of Concept and Application to Cluster Headache. Neurosurgery. 2016;79(6):806-14.

149. Concerto C, Al Sawah M, Chusid E, Trepal M, Taylor G, Aguglia E, et al. Anodal transcranial direct current stimulation for chronic pain in the elderly: a pilot study. Aging Clinical and Experimental Research. 2016;28(2):231-7.

150. Herrmann MJ, Beier JS, Simons B, Polak T. Transcranial direct current stimulation (tDCS) of the right inferior frontal gyrus attenuates skin conductance responses to unpredictable threat conditions. Frontiers in Human Neuroscience. 2016;10.

151. Kenter RM, Cuijpers P, Beekman A, van Straten A. Effectiveness of a Web-Based Guided Self-help Intervention for Outpatients With a Depressive Disorder: short-term Results From a Randomized Controlled Trial. Journal of medical Internet research. 2016;18(3):e80.

152. Khedr EM, Elbeh KAM, Elserogy Y, Khalifa HE, Ahmed MA, Hafez MH, et al. Motor cortical excitability in obsessive-compulsive disorder: Transcranial magnetic stimulation study. Neurophysiologie Clinique. 2016;46(2):135-43.

153. Khurshid K, Holbert R. Insomnia: A new approach to treatment with transcranial magnetic stimulation. Sleep. 2016;39:A214.

154. Leung A, Fallah A, Shukla S, Lin L, Tsia A, Song D, et al. RTMS in alleviating mild TBI related headaches – A case series. Pain Physician. 2016;19(2):E347-E53.

155. Malavera A, Silva FA, Fregni F, Carrillo S, Garcia RG. Repetitive Transcranial Magnetic Stimulation for Phantom Limb Pain in Land Mine Victims: A Double-Blinded, Randomized, Sham-Controlled Trial. The journal of pain. 2016;17(8):911-8.

156. Morin A, Léonard G, Gougeon V, Waddell G, Bureau YA, Girard I, et al. Efficacy of transcranial direct-current stimulation (tDCS) in women with provoked vestibulodynia: Study protocol for a randomized controlled trial. Trials. 2016;17(1).

157. Nct. Transcranial Direct Current Stimulation (tDCS) for Treatment-Resistant Obsessive-Compulsive Disorder (FRONT). https://clinicaltrialsgov/show/NCT02743715. 2016.

158. Nct. The Effect of Hypocaloric Diet Associated With tDCS on Weight Loss and Metabolic Profile. https://clinicaltrialsgov/show/NCT02683902. 2016.

159. Silva RM, Brunoni AR, Miguel EC, Shavitt RG. Transcranial direct current stimulation for treatment-resistant obsessive-compulsive disorder: report on two cases and proposal for a randomized, sham-controlled trial. Sao Paulo medical journal. 2016;134(5):446‐50.

160. Tanwar S, Mattoo B, Jain S, Kumar U, Dada R, Bhatia R. Transcranial magnetic stimulation in reducing chronic pain and the related symptoms in patients with fibromyalgia. Indian Journal of Physiology and Pharmacology. 2016;60(5):67-8.

161. Van 't Wout M, Mariano TY, Garnaat SL, Reddy MK, Rasmussen SA, Greenberg BD. Can Transcranial Direct Current Stimulation Augment Extinction of Conditioned Fear? Brain Stimulation. 2016;9(4):529-36.

162. Vennewald N, Winter B, Limburg K, Diemer J, Notzon S, Fohrbeck I, et al. Emotional processing and rTMS: does inhibitory theta burst stimulation affect the human startle reflex? Journal of Neural Transmission. 2016;123(10):1121-31.

163. Antal A, Alekseichuk I, Bikson M, Brockmöller J, Brunoni AR, Chen R, et al. Low intensity transcranial electric stimulation: Safety, ethical, legal regulatory and application guidelines. Clinical Neurophysiology. 2017;128(9):1774-809.

164. Ayache SS, Lefaucheur JP, Chalah MA. Long term effects of prefrontal tDCS on multiple sclerosis fatigue: A case study. Brain Stimulation. 2017;10(5):1001-2.

165. Bennebroek Evertsz F, Sprangers MAG, Sitnikova K, Stokkers PCF, Ponsioen CY, Bartelsman J, et al. Effectiveness of cognitive-behavioral therapy on quality of life, anxiety, and depressive symptoms among patients with inflammatory bowel disease: a multicenter randomized controlled trial. Journal of consulting and clinical psychology. 2017;85(9):918‐25.

166. Cachoeira CT, Leffa DT, Mittelstadt SD, Mendes LST, Brunoni AR, Pinto JV, et al. Positive effects of transcranial direct current stimulation in adult patients with attention-deficit/hyperactivity disorder – A pilot randomized controlled study. Psychiatry Research. 2017;247:28-32.

167. Caulfield KA, Bernstein MH, Stern AP, Pascual-Leone A, Press DZ, Fox MD. Antidepressant Effect of Low-Frequency Right-Sided rTMS in Two Patients with Left Frontal Stroke. Brain Stimulation. 2017;10(1):150-1.

168. Chalah MA, Lefaucheur JP, Créange A, Ayache SS. Targeting fatigue, mood and cognition in multiple sclerosis using tDCS. European Archives of Psychiatry and Clinical Neuroscience. 2017;267:S151-S2.

169. Chan W, Dobbs B, Shaw M, Kasschau M, Sherman K, Krupp L, et al. Baseline affect predicts improved fatigue with telerehabilitation using remotely-supervised transcranial direct current stimulation (RS-tDCS) in adults with multiple sclerosis (MS). Neurology. 2017;88(16).

170. Chang WH, Kim MS, Park E, Cho JW, Youn J, Kim YK, et al. Effect of Dual-Mode and Dual-Site Noninvasive Brain Stimulation on Freezing of Gait in Patients With Parkinson Disease. Archives of Physical Medicine and Rehabilitation. 2017;98(7):1283-90.

171. Cucca A, Migdadi H, Son AY, Gallo EC, Fisher SJ, Agarwal S, et al. Feasibility and safety of combining rtms with physical therapy: Preliminary data in parkinson's disease. Brain Stimulation. 2017;10(4):e63-e4.

172. Deppermann S, Vennewald N, Diemer J, Sickinger S, Haeussinger FB, Dresler T, et al. Neurobiological and clinical effects of fNIRS-controlled rTMS in patients with panic disorder/agoraphobia during cognitive-behavioural therapy. NeuroImage: Clinical. 2017;16:668-77.

173. Fallgatter AJ. Non-invasive brain stimulation-a modern approach to improved therapy of anxiety disorders? Brain stimulation. 2017;10(2):538‐.

174. Galea MP, Cofré Lizama LE, Bastani A, Panisset MG, Khan F. Cranial nerve non-invasive neuromodulation improves gait and balance in stroke survivors: A pilot randomised controlled trial. Brain Stimulation. 2017;10(6):1133-5.

175. Heeren A, Billieux J, Philippot P, De Raedt R, Baeken C, de Timary P, et al. Impact of transcranial direct current stimulation on attentional bias for threat: a proof-of-concept study among individuals with social anxiety disorder. Social cognitive and affective neuroscience. 2017;12(2):251‐60.

176. Herrmann MJ, Simons B, Polak T. Transcranial direct current stimulation (tDCS) of the right inferior frontal gyrus attenuates sustained fear. Brain stimulation. 2017;10(2):376‐.

177. Jeon H, Jang KI, Lee SH. Posttraumatic growth factor in baseline state predicts transcranial direct current stimulation treatment effects in patients with post-traumatic stress patients: An interim analysis. Brain Stimulation. 2017;10(2):433.

178. Kedzior KK, Müller C, Gerkensmeier I, Gellersen HM, Schuchinsky M. Predictors of clinical response to deep transcranial magnetic stimulation (dTMS) in unipolar major depression: A systematic review and meta-analysis. Brain Stimulation. 2017;10(2):354-5.

179. Knotkova H, Riggs A, Portenoy RK. A patient-tailored protocol of tDCS stimulation paired with telehealth support for at-home symptom management in seriously ill patients with multiple chronic symptoms. Brain Stimulation. 2017;10(4):e77-e8.

180. Kumar A, Bhatia R, Senthil S, Kumaran, Bhatia R. Targeting motor cortex with neuronavigated repetitive transcranial magnetic stimulation in management of chronic migraine. Indian Journal of Physiology and Pharmacology. 2017;61(5):115-6.

181. Kuo MF, Chen PS, Nitsche MA. The application of tDCS for the treatment of psychiatric diseases. International Review of Psychiatry. 2017;29(2):146-67.

182. Lattari E, Costa SS, Campos C, de Oliveira AJ, Machado S, Maranhao Neto GA. Can transcranial direct current stimulation on the dorsolateral prefrontal cortex improves balance and functional mobility in Parkinson's disease? Neuroscience Letters. 2017;636:165-9.

183. Lefebvre S, Liew SL. Anatomical parameters of tDCS to modulate the motor system after stroke: A review. Frontiers in Neurology. 2017;8(FEB).

184. Macaulay C, Angus L, Khattra J, Westra H, Ip J. Client Retrospective Accounts of Corrective Experiences in Motivational Interviewing Integrated With Cognitive Behavioral Therapy for Generalized Anxiety Disorder. Journal of clinical psychology. 2017;73(2):168‐81.

185. Marei A, Parmenter M, Rashed H, Pavlakis P, Holzberg S, Mona S, et al. Mood enhancement using repetitive transcranial magnetic stimulation (rTMS) to the left dorsolateral prefrontal cortex (LtDLPFC) as an adjuvant therapeutic technique may improve quality-of-life and disease progression. Amyotrophic Lateral Sclerosis and Frontotemporal Degeneration. 2017;18:232.

186. Nct. Transcranial Magnetic Stimulation for the Treatment of Veterans With Alcohol Use Disorders. https://clinicaltrialsgov/show/NCT03191266. 2017.

187. Palm U, Leitner B, Kirsch B, Behler N, Kumpf U, Wulf L, et al. Prefrontal tDCS and sertraline in obsessive compulsive disorder: a case report and review of the literature. Neurocase. 2017;23(2):173-7.

188. Sisko E, Rodriguez N, Corbett-Methot S, Sutton-DeBord J, Brown J, Tendler A. Multifocal dTMS to the dmPFC-ACC, bilateral PFC and insular cortices for highly resistant depression: Case report. Brain Stimulation. 2017;10(4):e36.

189. To WT, Ost J, Hart J, De Ridder D, Vanneste S. The added value of auditory cortex transcranial random noise stimulation (tRNS) after bifrontal transcranial direct current stimulation (tDCS) for tinnitus. Journal of Neural Transmission. 2017;124(1):79-88.

190. van‘t Wout M, Longo SM, Reddy MK, Philip NS, Bowker MT, Greenberg BD. Transcranial direct current stimulation may modulate extinction memory in posttraumatic stress disorder. Brain and Behavior. 2017;7(5).

191. van't Wout M, Longo SM, Reddy MK, Philip NS, Bowker MT, Greenberg BD. Transcranial direct current stimulation may modulate extinction memory in posttraumatic stress disorder. Brain and behavior. 2017;7(5).

192. Vlachos A. RTMS restores alterations in synaptic excitation/inhibitionbalance. European Archives of Psychiatry and Clinical Neuroscience. 2017;267:S140.

193. Augmentation of fear extinction by transcranial direct current stimulation (tDCS). Frontiers in behavioral neuroscience. 2018;12(no pagination).

194. Blumberger DM, Vila-Rodriguez F, Thorpe KE, Feffer K, Noda Y, Giacobbe P, et al. Effectiveness of theta burst versus high-frequency repetitive transcranial magnetic stimulation in patients with depression (THREE-D): a randomised non-inferiority trial. The Lancet. 2018;391(10131):1683-92.

195. Bocci T, De Carolis G, Mansani F, De Rosa A, Ferrucci R, Priori A, et al. Platform Session - NIBS: Cerebellar direct current stimulation (tDCS) for the treatment of phantom limb pain (PLP). Clinical Neurophysiology. 2018;129:e227.

196. Costanzo F, Menghini D, Maritato A, Castiglioni MC, Mereu A, Varuzza C, et al. New treatment perspectives in adolescents with anorexia nervosa: The efficacy of non-invasive brain-directed treatment. Frontiers in Behavioral Neuroscience. 2018;12.

197. De Hemptinne C, Chen W, Racine C, Seritan A, Miller A, Mergenthaler J, et al. Prefrontal cortical stimulation to treat anxiety and depression in Parkinson's disease. Movement Disorders. 2018;33:S802.

198. Dittert N, Hüttner S, Polak T, Herrmann MJ. Augmentation of fear extinction by transcranial direct current stimulation (tDCS). Frontiers in Behavioral Neuroscience. 2018;12.

199. Donse L, Padberg F, Sack AT, Rush AJ, Arns M. Simultaneous rTMS and psychotherapy in major depressive disorder: Clinical outcomes and predictors from a large naturalistic study. Brain Stimulation. 2018;11(2):337-45.

200. Drks. Transdiagnostic internet-based and mobile-supported intervention for the treatment of anxiety disorders. http://wwwwhoint/trialsearch/Trial2aspx?TrialID=DRKS00012656. 2018.

201. Dutt-Mazumder A, Brown S, Dharia A, Vogel A, Talati R, Gardi A, et al. Effects of different transcranial direct current stimulation devices on motor cortical excitability. Neurorehabilitation and neural repair. 2018;32(12):1096‐7.

202. Fitzgibbon BM, Hoy KE, Knox LA, Guymer EK, Littlejohn G, Elliot D, et al. Evidence for the improvement of fatigue in fibromyalgia: A 4-week left dorsolateral prefrontal cortex repetitive transcranial magnetic stimulation randomized-controlled trial. European Journal of Pain (United Kingdom). 2018;22(7):1255-67.

203. Güleken Z, Eskikurt G, Karamürsel S. Investigation of the effects of transcranial direct current stimulation and neurofeedback by continuous performance test and beck inventories. Clinical Neurophysiology. 2018;129:e127-e8.

204. Hosseinzadeh SA, Mazhari S, Najafi K, Ahmadi M, Aghaei I, Khaksarian M. Anodal transcranial direct current stimulation enhances positive changes in movement functions, visual attention and depression of patients with chronic ischemic stroke: A clinical trial. Biomedical Research and Therapy. 2018;5(11):2841-9.

205. Iznak A, Iznak E, Oleichik I, Medvedeva T, Damyanovich E. Clinical, psychological and EEG effects of combined treatment of pharmaevresistant depression included rhythmic transcranial magnetic stimulation. European Psychiatry. 2018;48:S509.

206. Jprn U. Randomized Controlled Trial of Cognitive-Behavioral Therapy versus Mindfulness-Based Intervention for Depression in University Students. http://wwwwhoint/trialsearch/Trial2aspx?TrialID=JPRN-UMIN000032736. 2018.

207. Lagueux E, Bernier M, Bourgault P, Whittingstall K, Mercier C, Léonard G, et al. The Effectiveness of Transcranial Direct Current Stimulation as an Add-on Modality to Graded Motor Imagery for Treatment of Complex Regional Pain Syndrome A Randomized Proof of Concept Study. Clinical Journal of Pain. 2018;34(2):145-54.

208. Lanza G, Bella R, Cantone M, Pennisi G, Ferri R, Pennisi M. Cognitive impairment and celiac disease: Is transcranial magnetic stimulation a Trait d’Union between gut and brain? International Journal of Molecular Sciences. 2018;19(8).

209. Lattari E, de Oliveira BS, Oliveira BRR, de Mello Pedreiro RC, Machado S, Neto GAM. Effects of transcranial direct current stimulation on time limit and ratings of perceived exertion in physically active women. Neuroscience Letters. 2018;662:12-6.

210. Margolis SA, Festa EK, Korthauer LE, Gonsalves M, Oberman LM, Heindel WC, et al. REPETITIVE TRANSCRANIAL MAGNETIC STIMULATION IN PRIMARY PROGRESSIVE APHASIAS AND BEHAVIORAL VARIANT FRONTOTEMPORAL DEMENTIA: A CASE SERIES. Alzheimer's and Dementia. 2018;14(7):P675.

211. Mariano T, Burgess F, Bowker M, Kirschner J, Van 'T Wout-Frank M, Halladay C, et al. Transcranial direct current stimulation (TDCS) for the affective symptoms of chronic low back pain (CLBP): a double-blinded, randomized, placebo-controlled trial. Biological psychiatry. 2018;83(9):S200‐S1.

212. Nct. Efficacy of Repetitive Transcranial Magnetic Stimulation for Improvement of Memory in Older Adults With TBI. https://clinicaltrialsgov/show/NCT03727737. 2018.

213. Nct. Enhancement of Therapeutic Learning in OCD Using tDCS. https://clinicaltrialsgov/show/NCT03572543. 2018.

214. Nct. Cycloserine rTMS Plasticity Augmentation. https://clinicaltrialsgov/show/NCT03432689. 2018.

215. O'Toole MS, Watson L, Rosenberg NK, Berntsen D. Changes in perceived centrality of anxious events following cognitive behavioral therapy for social anxiety disorder and panic disorder. Journal of Behavior Therapy and Experimental Psychiatry. 2018;59:150-6.

216. Ottaviani C, Mancini F, Provenzano S, Collazzoni A, D'Olimpio F. Deontological morality can be experimentally enhanced by increasing disgust: a transcranial direct current stimulation study. Neuropsychologia. 2018;119:474‐81.

217. Phillips AL, Burr RL, Dunner DL. Management of treatment resistant bipolar depression with rTMS. Brain Stimulation. 2018;11(6):e10.

218. Sauvaget A, Bulteau S, Guilleux A, Leboucher J, Pichot A, Valrivière P, et al. Both active and sham low-frequency rTMS single sessions over the right DLPFC decrease cue-induced cravings among pathological gamblers seeking treatment: A randomized, double-blind, sham-controlled crossover trial. Journal of Behavioral Addictions. 2018;7(1):126-36.

219. Shahbabaie A, Hatami J, Farhoudian A, Ekhtiari H, Khatibi A, Nitsche MA. Optimizing electrode montages of transcranial direct current stimulation for attentional bias modification in early abstinent methamphetamine users. Frontiers in Pharmacology. 2018;9(AUG).

220. Siebmanns S, Ulander M, Sandberg J, Johansson L, Johansson P, Broström A. Internet-based CBT for insomnia in the general population-a description of design, measurements and interventions in recent RCT studies. Journal of Sleep Research. 2018;27:290-1.

221. Tctr. Comparison of the mindfulness and transcranial stimulation on brain injury. http://wwwwhoint/trialsearch/Trial2aspx?TrialID=TCTR20180827003. 2018.

222. Weisel KK, Zarski AC, Berger T, Krieger T, Schaub MP, Moser CT, et al. Efficacy and cost-effectiveness of guided and unguided internet- and mobile-based indicated transdiagnostic prevention of depression and anxiety (ICare Prevent): a three-armed randomized controlled trial in four European countries. Internet interventions. 2018.

223. xnjbc RBR. Non-Invasive Brain Stimulation in the treatment of Pain due to brachial plexus injury. http://wwwwhoint/trialsearch/Trial2aspx?TrialID=RBR-5xnjbc. 2018.

224. Ahmed M, Siddiqi S, Haque S, Chau J, Amin H. Safety and efficacy of repetitive Transcranial Magnetic Stimulation (rTMS) for depression in mild to moderate Traumatic Brain Injury (TBI): a non-controlled study. Brain Stimulation. 2019;12(2):585.

225. Aisenberg Romano G, Fried Zaig I, Halevy A, Azem F, Amit A, Bloch M. Prophylactic SSRI treatment for women suffering from mood and anxiety symptoms undergoing in vitro fertilization-a prospective placebo-controlled study. Archives of women's mental health. 2019;22(4):503‐10.

226. Arango-Gutiérrez AS, Buitrago-Cifuentes LJ, Medina-Hinestroza AM, Molina-Paniagua SA, Moreno E, Rivera-Díaz JS, et al. Sonotherapy in the reduction of anxiety and postoperative pain in patients with regional anesthesia as a sole technique: Randomized, controlled clinical trial. Cirugia y Cirujanos (English Edition). 2019;87(5):545-53.

227. Balderston N, Roberts C, Masi E, Deng ZD, Radman T, Luber B, et al. Repetitive Transcranial Magnetic Stimulation Reveals a Causal Link Between Right dlPFC Activity and Anxiety Expression. Biological Psychiatry. 2019;85(10):S135.

228. Barredo J, Swearingen H, Greenberg B, Carpenter L, Philip N. Neuroimaging of transcranial magnetic stimulation for suicidality. Neuropsychopharmacology. 2019;44:263-4.

229. Bocci T, De Carolis G, Mansani F, Ferrucci R, Priori A, Valeriani M, et al. Cerebellar Direct Current Stimulation (ctDCS) for the treatment of painful and non painful phantom limb phenomena (PLP). Brain Stimulation. 2019;12(2):444-5.

230. Bonello M, Nurmikko T, Mavrianou A, Steiger M. Repetitive transcranial magnetic stimulation (rTMS) for the treatment of pain in Parkinson's Disease-an open-label study. Movement disorder. 2019;34:S624‐S6.

231. Carnevali L, Pattini E, Sgoifo A, Ottaviani C. Effects of prefrontal transcranial direct current stimulation on autonomic and neuroendocrine responses to psychosocial stress in healthy humans. Stress (Amsterdam, Netherlands). 2019:1‐11.

232. Cheng JY, Wang Z. Progress of transcranial direct current stimulation for treatment of obsessive-compulsive disorder. Journal of Shanghai Jiaotong University (Medical Science). 2019;39(9):1089-94.

233. Da Silva RMF, Brunoni AR, Miguel EC, Shavitt RG. Transcranial direct current stimulation for obsessive- compulsive disorder: Patient selection and perspectives. Neuropsychiatric Disease and Treatment. 2019;15:2663-9.

234. de Lima AL, Braga FMA, da Costa RMM, Gomes EP, Brunoni AR, Pegado R. Transcranial direct current stimulation for the treatment of generalized anxiety disorder: a randomized clinical trial. Journal of affective disorders. 2019;259:31‐7.

235. De Witte S, Pulopulos MM, Vanderhasselt M, De Raedt R, Schiettecatte J, Anckaert E, et al. Individual differences in state anxiety influence the effect of iTBS over the left dorsolateral prefrontal cortex on HPA sensitivity. Brain Stimulation. 2019;12(2):425.

236. Deng H, Cole E, Gulser M, Stimpson K, Tischler C, Sudheimer K, et al. Depressive symptoms improved by accelerated intermittent theta-burst stimulation. Brain Stimulation. 2019;12(2):480.

237. Fountoulakis KN, Karavelas V, Moysidou S, Mavridis D, Pastiadis K, Petalidou N, et al. Efficacy of Add-on Pregabalin in the Treatment of Patients with Generalized Anxiety Disorder and Unipolar Major Depression With an Early Nonresponse to Escitalopram: a Double-Blind Placebo-Controlled Study. Pharmacopsychiatry. 2019;52(4):193‐202.

238. Fricová J, Englerová K, Nedvídek J, Rokyta R. The treatment of orofacial pain by using transcranial direct current stimulation. Physiological research. 2019;68:S367-S72.

239. Galhardoni R, Aparecida da Silva V, García-Larrea L, Dale C, Baptista AF, Barbosa LM, et al. Insular and anterior cingulate cortex deep stimulation for central neuropathic pain: Disassembling the percept of pain. Neurology. 2019;92(18):e2165-e75.

240. Gold AK, Ornelas AC, Cirillo P, Caldieraro MA, Nardi AE, Nierenberg AA, et al. Clinical applications of transcranial magnetic stimulation in bipolar disorder. Brain and Behavior. 2019;9(10).

241. Herrmann M, Simons B, Horst A, Boehme S, Straube T, Polak T. Transcranial direct current stimulation (tDCS) of the right inferior frontal cortex (rIFC) attenuates sustained fear. Brain stimulation. 2019;12(2):432‐.

242. Js G, E J, Ja T, Kh B, Kc O, Gover-Chamlou A, et al. Evolution of a Study of Bilateral Prefrontal Transcranial Magnetic Stimulation (TMS) to Treat the Symptoms of Mild TBI (mTBI) and PTSD: research Methods, Participant Demographics, and Tolerability. Brain stimulation. 2019;12(4):e135‐.

243. Kaster TS, Downar J, Vila-Rodriguez F, Thorpe KE, Feffer K, Noda Y, et al. Trajectories of response to dorsolateral prefrontal rTMS in major depression: A three-D study. American Journal of Psychiatry. 2019;176(5):367-75.

244. Lee J, Kim S, Chang H, Oh E, Sohn E, Lee A. Experience of repetitive transcranial magnetic stimulation in severe dementia with hereditary diffuse leukoencephalopathy with axonal spheroid. Brain Stimulation. 2019;12(2):575-6.

245. m5j4s RBR. Non-Invasive Brain Stimulation Associated with Aerobic Exercise: repercussions on Pain, Memory, and Sleep of Individuals with Chronic Migraine. http://wwwwhoint/trialsearch/Trial2aspx?TrialID=RBR-4m5j4s. 2019.

246. Mariano TY, Burgess FW, Bowker M, Kirschner J, van’t Wout-Frank M, Jones RN, et al. Transcranial direct current stimulation for affective symptoms and functioning in chronic low back pain: A pilot double-blinded, randomized, placebo-controlled trial. Pain Medicine (United States). 2019;20(6):1166-77.

247. Marques RC, Vieira L, Marques D, Cantilino A. Transcranial magnetic stimulation of the medial prefrontal cortex for psychiatric disorders: A systematic review. Brazilian Journal of Psychiatry. 2019;41(5):447-57.

248. Martinotti G, Lupi M, Montemitro C, Miuli A, Di Natale C, Spano MC, et al. Transcranial Direct Current Stimulation Reduces Craving in Substance Use Disorders: A Double-blind, Placebo-Controlled Study. Journal of ECT. 2019;35(3):207-11.

249. Martinotti G, Miuli A, Sepede G, Di Natale C, Spano M, Lorusso M, et al. Transcranial direct current stimulation in cocaine use disorders: preliminary findings. Brain Stimulation. 2019;12(2):489.

250. McLaughlin N, Barredo J, Blanchette B, Carpenter L, Philip N, Phillips M, et al. Cathodal transcranial direct current stimulation targeting the pre-supplementary motor area on resting state functional connectivity in OCD. Neuropsychopharmacology. 2019;44:161.

251. Mehrsafar AH, Gazerani P. Non-invasive brain stimulation in athletic competition. Apunts Medicina de l'Esport. 2019;54(203):105-6.

252. Nct. The Effect Of Transcranial Direct Current Stimulation In Panic Disorder. https://clinicaltrialsgov/show/NCT04160806. 2019.

253. Nct. Transcranial Direct Current Stimulation and Fear Extinction. https://clinicaltrialsgov/show/NCT03907917. 2019.

254. Nct. Augmentation of EMDR With tDCS in the Treatment of Fibromyalgia. https://clinicaltrialsgov/show/NCT04084795. 2019.

255. Parlikar R, Sreeraj VS, Chhabra H, Thimmashetty VH, Parameshwaran S, Selvaraj S, et al. Add-on HD-tDCS for obsessive-compulsive disorder with comorbid bipolar affective disorder: A case series. Asian Journal of Psychiatry. 2019;43:87-90.

256. Patel S, Lavrador J, Ghimire P, Gullan R, Ashkan K, Bhangoo R, et al. Patient reported experience measures in navigated transcranial magnetic stimulation. Neuro-Oncology. 2019;21:iv15.

257. Philip NS, Leuchter AF, Cook IA, Massaro J, Goethe JW, Carpenter LL. Predictors of response to synchronized transcranial magnetic stimulation for major depressive disorder. Depression and anxiety. 2019;36(3):278‐85.

258. Poppa Fioretti T, de Witte S, Vanderhasselt M, Bechara A, Baeken C. Theta-burst stimulation and prefrontal regulation of cardiovascular autonomic outputs: the role of state anxiety. Brain Stimulation. 2019;12(2):510.

259. Rothärmel M, Moulier V, Vasse M, Isaac C, Faerber M, Bendib B, et al. A Prospective Open-Label Pilot Study of Transcranial Direct Current Stimulation in High-Functioning Autistic Patients with a Dysexecutive Syndrome. Neuropsychobiology. 2019;78(4):189-99.

260. Scarpino M, Lanzo G, Salimova M, Lolli F, del Vecchio A, Cossu C, et al. Efficacy of high-frequency (15 Hz) repetitive transcranial magnetic stimulation (rTMS) of the left premotor cortex/dorsolateral prefrontal cortex in decreasing cocaine intake (the MagneTox study): A study protocol for a randomized placebo-controlled pilot trial. Neurophysiologie Clinique. 2019;49(1):1-9.

261. Singh A, Cao S, Kingyon J, Parker KL. Proceedings #32: effects of cerebellar delta and theta frequency tACS on cognitive performance in patients with schizophrenia. Brain stimulation. 2019;12(2):e99‐e100.

262. Song P, Lin H, Li S, Wang L, Liu J, Li N, et al. Repetitive transcranial magnetic stimulation (rTMS)modulates time-varying electroencephalography (EEG)network in primary insomnia patients: a TMS-EEG study. Sleep Medicine. 2019;56:157-63.

263. Wang S, Sun Q, Zhai L, Bai Y, Wei W, Jia L. The prevalence of depression and anxiety symptoms among overweight/obese and non-overweight/non-obese children/adolescents in China: A systematic review and meta-analysis. International Journal of Environmental Research and Public Health. 2019;16(3).

264. Zandvakili A, Berlow YA, Carpenter LL, Philip NS. Transcranial direct current stimulation in psychiatry: What psychiatrists need to know. Focus (United States). 2019;17(1):44-9.

265. Zheng Y, Zhong D, Huang Y, He M, Xiao Q, Jin R, et al. Effectiveness and safety of repetitive transcranial magnetic stimulation (rTMS) on aphasia in cerebrovascular accident patients: Protocol of a systematic review and meta-analysis. Medicine (United States). 2019;98(52).

266. Zorzo C, Banqueri M, Higarza SG, Pernía AM, Arias JL. Current State of Transcranial Magnetic Stimulation and its use in Psychiatry. Actas espanolas de psiquiatria. 2019;47(3):110-20.

267. Non-invasive brain stimulation for modulating a consolidated fear memory. Pharmacopsychiatry. 2020;53(2):92‐.

268. Low-frequency parietal repetitive transcranial magnetic stimulation reduces fear and anxiety. Translational psychiatry. 2020;10(1).

269. Investigation of the effects of transcranial direct current stimulation and neurofeedback by continuous performance test. Neuroscience letters. 2020;716.

270. Actrn. Using mild non-invasive brain stimulation for identifying and targeting brain-based changes in anxiety. http://wwwwhoint/trialsearch/Trial2aspx?TrialID=ACTRN12620000558921. 2020.

271. Actrn. A multisite clinical trial of repetitive transcranial magnetic stimulation (rTMS) for social communication in autism spectrum disorder (ASD). http://wwwwhoint/trialsearch/Trial2aspx?TrialID=ACTRN12620000890932. 2020.

272. Ayache SS, Riachi N, Ahdab R, Chalah MA. Effects of transcranial direct current stimulation on hand dexterity in multiple sclerosis: a design for a randomized controlled trial. Brain sciences. 2020;10(3).

273. Balderston NL, Beydler EM, Goodwin M, Deng ZD, Radman T, Luber B, et al. Low-frequency parietal repetitive transcranial magnetic stimulation reduces fear and anxiety. Translational Psychiatry. 2020;10(1).

274. Balderston NL, Beydler EM, Roberts C, Deng ZD, Radman T, Lago T, et al. Mechanistic link between right prefrontal cortical activity and anxious arousal revealed using transcranial magnetic stimulation in healthy subjects. Neuropsychopharmacology. 2020;45(4):694-702.

275. Balderston NL, Roberts C, Beydler EM, Deng ZD, Radman T, Luber B, et al. A generalized workflow for conducting electric field–optimized, fMRI-guided, transcranial magnetic stimulation. Nature Protocols. 2020;15(11):3595-614.

276. Caulfield KA, Stern AP. Therapeutic High-Frequency Repetitive Transcranial Magnetic Stimulation Concurrently Improves Mood and Anxiety in Patients Using Benzodiazepines. Neuromodulation. 2020;23(3):380-3.

277. Chalah MA, Grigorescu C, Padberg F, Kumpfel T, Palm U, Ayache SS. Bifrontal transcranial direct current stimulation modulates fatigue in multiple sclerosis: a randomized sham-controlled study. Journal of neural transmission. 2020.

278. Dong W, Luo B, Qiu C, Jiang X, Qu X, Zhang L, et al. Deep Brain Stimulation for the Treatment of Dopa-Responsive Dystonia: A Case Report and Literature Review. World Neurosurgery. 2020;136:394-8.e5.

279. Dutra LRDV, Pegado R, Silva LK, Dantas HS, Câmara HA, Silva-Filho EM, et al. Modulating anxiety and functional capacity with anodal tDCS over the left dorsolateral prefrontal cortex in primary dysmenorrhea. International Journal of Women's Health. 2020;12:243-51.

280. Esposito M, Ferrari C, Fracassi C, Miniussi C, Brignani D. Baseline levels of arousal are crucial in predicting tDCS effects. Clinical Neurophysiology. 2020;131(4):e87.

281. Green PE, Loftus A, Anderson RA. Protocol for transcranial direct current stimulation for obsessive-compulsive disorder. Brain sciences. 2020;10(12):1‐9.

282. Guleken Z, Eskikurt G, Karamürsel S. Investigation of the effects of transcranial direct current stimulation and neurofeedback by continuous performance test. Neuroscience Letters. 2020;716.

283. Hadipour AL, Vrticka P, Kazemi R, Nasiri Z. The effect of a single session of high frequency rTMS on heart rate variability; an investigation of the role of RDLPFC in emotional appraisal and regulation. Clinical Neurophysiology. 2020;131(4):e28.

284. Hett D, Marwaha S. Repetitive Transcranial Magnetic Stimulation in the Treatment of Bipolar Disorder. Therapeutic Advances in Psychopharmacology. 2020;10.

285. Lefaucheur JP, Aleman A, Baeken C, Benninger DH, Brunelin J, Di Lazzaro V, et al. Evidence-based guidelines on the therapeutic use of repetitive transcranial magnetic stimulation (rTMS): An update (2014–2018). Clinical Neurophysiology. 2020;131(2):474-528.

286. Leong K, Chan P, Ong L, Zwicker A, Willan S, Lam RW, et al. A Randomized Sham-controlled Trial of 1-Hz and 10-Hz Repetitive Transcranial Magnetic Stimulation (rTMS) of the Right Dorsolateral Prefrontal Cortex in Civilian Post-traumatic Stress Disorder: Un essai randomisé contrôlé simulé de stimulation magnétique transcrânienne repetitive (SMTr) de 1 Hz et 10 Hz du cortex préfrontal dorsolatéral droit dans le trouble de stress post-traumatique chez des civils. Canadian Journal of Psychiatry. 2020;65(11):770-8.

287. Liang JN, Ubalde L, Jacklin J, Hobson P, Wright-Avila S, Lee YJ. Immediate Effects of Anodal Transcranial Direct Current Stimulation on Postural Stability Using Computerized Dynamic Posturography in People With Chronic Post-stroke Hemiparesis. Frontiers in Human Neuroscience. 2020;14.

288. Lin Y, Chen P, Yang K, Zhou Q, Wang Y. Efficacy of repetitive dual-site paired associative transcranial magnetic stimulation in the treatment of generalized anxiety disorder. Clinical Neurophysiology. 2020;131(4):e15-e6.

289. McClintock SM, Martin DM, Lisanby SH, Alonzo A, McDonald WM, Aaronson ST, et al. Neurocognitive effects of transcranial direct current stimulation (tDCS) in unipolar and bipolar depression: Findings from an international randomized controlled trial. Depression and Anxiety. 2020;37(3):261-72.

290. Nct. Efficacy of Clinical Application of Transcranial Low Intensity Focused Ultrasonic Stimulation for Patients With Major Deperessive Disorder - Exploratory Clinical Trial. https://clinicaltrialsgov/show/NCT04405791. 2020.

291. Nct. tDCS for Food-related Impulsivity in Obesity. https://clinicaltrialsgov/show/NCT04218383. 2020.

292. Nct. Accelerated Bilateral rTMS on Geriatric Depression. https://clinicaltrialsgov/show/NCT04486222. 2020.

293. Nct. tDCS and Motor Learning in Children With DCD. https://clinicaltrialsgov/show/NCT04490187. 2020.

294. Nct. Home-Based tDCS in Children With ADHD: a Randomized, Sham-Controlled tDCS and fNIRS Study. https://clinicaltrialsgov/show/NCT04634006. 2020.

295. Nct. Roflumilast TMS-EEG Plasticity. https://clinicaltrialsgov/show/NCT04369547. 2020.

296. Nct. tDCS and Pain Associated With Diabetic Neuropathy. https://clinicaltrialsgov/show/NCT04306289. 2020.

297. Orta DSJ, May-Mas RN, Quiñones-Pesqueira GA, Diaz-Victoria AR, Gutierrez-Soriano CB, Tellez-Silva M, et al. Effects and safety of the anodal transcraneal direct current electrical stimulation in the cognitive profile of medical residents with acute sleep deprivation. Journal of Clinical Neurophysiology. 2020;37(4):370-1.

298. Overman MJ, Browning M, O’Shea J. Inducing Affective Learning Biases with Cognitive Training and Prefrontal tDCS: A Proof-of-Concept Study. Cognitive Therapy and Research. 2020.

299. Palm U, Brunelin J, Wulf L, Mondino M, Brunoni AR, Padberg F. Transcranial direct current stimulation (tDCS) for obsessive-compulsive disorder: A new treatment option? Fortschritte der Neurologie Psychiatrie. 2020;88(7):451-8.

300. Patel S, Ghimire P, Lavrador JP, Jung J, Gullan R, Ashkan K, et al. Patient-reported experience measures in patients undergoing navigated transcranial magnetic stimulation (nTMS): the introduction of nTMS-PREMs. Acta Neurochirurgica. 2020;162(7):1673-81.

301. Phillips AL, Burr RL, Dunner DL. Repetitive transcranial magnetic stimulation in the treatment of bipolar depression: Experience from a clinical setting. Journal of Psychiatric Practice. 2020;26(1):37-45.

302. Poppa T, de Witte S, Vanderhasselt MA, Bechara A, Baeken C. Theta-burst stimulation and frontotemporal regulation of cardiovascular autonomic outputs: The role of state anxiety. International Journal of Psychophysiology. 2020;149:25-34.

303. Shiasy Y, Shakiba S, Taremian F, Akhavan Hejazi SM, Abasi A. The effectiveness of attention bias modification with and without trans cranial direct current stimulation in chronic low back pain. Iranian Journal of Psychiatry. 2020;15(2):112-25.

304. Singh H, Neil LA. Incidence of side effects in patients receiving Repetitive Transcranial Magnetic Stimulation (rTMS). Brain Stimulation. 2020;13(6):1847-8.

305. Smits F, Geuze E, Gladwin T. Can tDCS improve impulse control and symptoms of PTSD, anxiety and aggression in military personnel and veterans? Clinical Neurophysiology. 2020;131(4):e60-e1.

306. Stein DJ, Medeiros LF, Caumo W, Torres ILS. Transcranial direct current stimulation in patients with anxiety: Current perspectives. Neuropsychiatric Disease and Treatment. 2020;16:161-9.

307. Stilling J, Paxman E, Mercier L, Gan LS, Wang M, Amoozegar F, et al. Treatment of Persistent Post-Traumatic Headache and Post-Concussion Symptoms Using Repetitive Transcranial Magnetic Stimulation: A Pilot, Double-Blind, Randomized Controlled Trial. Journal of Neurotrauma. 2020;37(2):312-23.

308. Van Lieshout ECC, Jacobs LD, Pelsma M, Dijkhuizen RM, Visser-Meily JMA. Exploring the experiences of stroke patients treated with transcranial magnetic stimulation for upper limb recovery: A qualitative study. BMC Neurology. 2020;20(1).

309. Acceptability of tDCS in treating stress-related mental health disorders: a mixed methods study among military patients and caregivers. BMC psychiatry. 2021;21(1).

310. Bernardi L, Bertuccelli M, Formaggio E, Rubega M, Bosco G, Tenconi E, et al. Beyond physiotherapy and pharmacological treatment for fibromyalgia syndrome: tailored tACS as a new therapeutic tool. European Archives of Psychiatry and Clinical Neuroscience. 2021;271(1):199-210.

311. Bulubas L, Padberg F, Mezger E, Suen P, Bueno PV, Duran F, et al. Prefrontal resting-state connectivity and antidepressant response: no associations in the ELECT-TDCS trial. European Archives of Psychiatry and Clinical Neuroscience. 2021;271(1):123-34.

312. Forogh B, Haqiqatshenas H, Ahadi T, Ebadi S, Alishahi V, Sajadi S. Repetitive transcranial magnetic stimulation (rTMS) versus transcranial direct current stimulation (tDCS) in the management of patients with fibromyalgia: A randomized controlled trial. Neurophysiologie Clinique. 2021.

313. Gardoki-Souto I, Martín de la Torre O, Hogg B, Redolar-Ripoll D, Valiente-Gómez A, Martínez Sadurní L, et al. Augmentation of EMDR with multifocal transcranial current stimulation (MtCS) in the treatment of fibromyalgia: study protocol of a double-blind randomized controlled exploratory and pragmatic trial. Trials. 2021;22(1).

314. Goerigk SA, Padberg F, Chekroud A, Kambeitz J, Bühner M, Brunoni AR. Parsing the antidepressant effects of non-invasive brain stimulation and pharmacotherapy: A symptom clustering approach on ELECT-TDCS. Brain Stimulation. 2021;14(4):906-12.

315. González-Zamorano Y, Fernández-Carnero J, Sánchez-Cuesta FJ, Arroyo-Ferrer A, Vourvopoulos A, Figueiredo P, et al. New approaches based on non-invasive brain stimulation and mental representation techniques targeting pain in parkinson’s disease patients: Two study protocols for two randomized controlled trials. Brain Sciences. 2021;11(1):1-14.

316. Isrctn. Sertraline for anxiety in adults with a diagnosis of autism. http://wwwwhoint/trialsearch/Trial2aspx?TrialID=ISRCTN15984604. 2021.

317. Kumar A, Mattoo B, Bhatia R, Kumaran S, Bhatia R. Neuronavigation based 10 sessions of repetitive transcranial magnetic stimulation therapy in chronic migraine: an exploratory study. Neurological Sciences. 2021;42(1):131-9.

318. McGirr A, Vila-Rodriguez F, Cole J, Torres IJ, Arumugham SS, Keramatian K, et al. Efficacy of Active vs Sham Intermittent Theta Burst Transcranial Magnetic Stimulation for Patients with Bipolar Depression: A Randomized Clinical Trial. JAMA Network Open. 2021;4(3).

319. McPhee ME, Graven-Nielsen T. Medial Prefrontal High-Definition Transcranial Direct Current Stimulation to Improve Pain Modulation in Chronic Low Back Pain: A Pilot Randomized Double-blinded Placebo-Controlled Crossover Trial. Journal of Pain. 2021.

320. Miron JP, Voetterl H, Fox L, Hyde M, Mansouri F, Dees S, et al. Optimized repetitive transcranial magnetic stimulation techniques for the treatment of major depression: A proof of concept study. Psychiatry Research. 2021;298.

321. Mukhdomi T, Mukhdomi J, Harris M, Sidhom P, Whitten I, Fragoza K, et al. Reviewing Complications Associated with Cranial Electrical Stimulation, an FDA MAUDE Database Investigation. Neuromodulation. 2021;24(4):e8.

322. Nct. Psychotherapy of Anxiety Disorders With Noninvasive Brain Stimulation - Using Virtual Reality. https://clinicaltrialsgov/show/NCT04782570. 2021.

323. Nct. Effects of Intravenous (IV) Citalopram Hydrochloride During Transcranial Magnetic Stimulation in Major Depressive Disorder (MDD). https://clinicaltrialsgov/show/NCT04846829. 2021.

324. Oathes DJ, Zimmerman JP, Duprat R, Japp SS, Scully M, Rosenberg BM, et al. Resting fMRI-guided TMS results in subcortical and brain network modulation indexed by interleaved TMS/fMRI. Experimental Brain Research. 2021;239(4):1165-78.

325. Overvliet GM, Jansen RAC, van Balkom AJLM, van Campen DC, Oudega ML, van der Werf YD, et al. Adverse events of repetitive transcranial magnetic stimulation in older adults with depression, a systematic review of the literature. International Journal of Geriatric Psychiatry. 2021;36(3):383-92.

326. Silva RMF, Brunoni AR, Goerigk S, Batistuzzo MC, Costa DLC, Diniz JB, et al. Efficacy and safety of transcranial direct current stimulation as an add-on treatment for obsessive-compulsive disorder: a randomized, sham-controlled trial. Neuropsychopharmacology. 2021;46(5):1028-34.

327. Zhao CG, Sun W, Ju F, Jiang S, Wang H, Sun XL, et al. Analgesic Effects of Navigated Repetitive Transcranial Magnetic Stimulation in Patients With Acute Central Poststroke Pain. Pain and therapy. 2021.

**Not RCT(n = 51)**

1. Boldt I, Eriks-Hoogland I, Brinkhof MW, de Bie R, Joggi D, von Elm E. Non-pharmacological interventions for chronic pain in people with spinal cord injury. Cochrane Database of Systematic Reviews. (11):CD009177.

2. Bystritsky A, Kerwin L, Feusner J. A pilot study of cranial electrotherapy stimulation for generalized anxiety disorder. Journal of Clinical Psychiatry.69(3):412-7.

3. Gilula MF. Cranial electrotherapy stimulation and fibromyalgia. Expert Review of Medical Devices.4(4):489-95.

4. Gilula MF, Barach PR. Cranial electrotherapy stimulation: a safe neuromedical treatment for anxiety, depression, or insomnia. Southern Medical Journal.97(12):1269-70.

5. Kirsch DL, Nichols F. Cranial electrotherapy stimulation for treatment of anxiety, depression, and insomnia. Psychiatric Clinics of North America.36(1):169-76.

6. Kittel-Schneider S, Reif A. [Treatment of psychiatric disorders during pregnancy and the breast feeding : Psychotherapy and other nondrug therapies]. Nervenarzt.87(9):967-73.

7. Klawansky S, Yeung A, Berkey C, Shah N, Phan H, Chalmers TC. Meta-analysis of randomized controlled trials of cranial electrostimulation. Efficacy in treating selected psychological and physiological conditions. Journal of Nervous & Mental Disease.183(7):478-84.

8. Luckett T, Butow PN, King MT, Oguchi M, Heading G, Hackl NA, et al. A review and recommendations for optimal outcome measures of anxiety, depression and general distress in studies evaluating psychosocial interventions for English-speaking adults with heterogeneous cancer diagnoses. Supportive Care in Cancer.18(10):1241-62.

9. Nugent K, Cevik C. Use and safety of conducted electronic devices: what is known? Eastern Mediterranean Health Journal.19(6):576-9.

10. Roy-Byrne P. Treatment-refractory anxiety; definition, risk factors, and treatment challenges. Dialogues in Clinical Neuroscience.17(2):191-206.

11. Sathappan AV, Luber BM, Lisanby SH. The Dynamic Duo: Combining noninvasive brain stimulation with cognitive interventions. Progress in Neuro-Psychopharmacology & Biological Psychiatry.89:347-60.

12. Shekelle PG, Cook IA, Miake-Lye IM, Booth MS, Beroes JM, Mak S. Benefits and Harms of Cranial Electrical Stimulation for Chronic Painful Conditions, Depression, Anxiety, and Insomnia: A Systematic Review. Annals of Internal Medicine.168(6):414-21.

13. Yennurajalingam S, Kang DH, Hwu WJ, Padhye NS, Masino C, Dibaj SS, et al. Cranial Electrotherapy Stimulation for the Management of Depression, Anxiety, Sleep Disturbance, and Pain in Patients With Advanced Cancer: A Preliminary Study. Journal of Pain & Symptom Management.55(2):198-206.

14. Yu B, Qiu H, Li J, Zhong C, Li J. Noninvasive Brain Stimulation Does Not Improve Neuropathic Pain in Individuals With Spinal Cord Injury: Evidence From a Meta-Analysis of 11 Randomized Controlled Trials. American Journal of Physical Medicine & Rehabilitation.99(9):811-20.

15. Klawansky S, Yeung A, Berkey C, Shah N, Phan H, Chalmers TC. Meta-analysis of randomized controlled trials of cranial electrostimulation. Efficacy in treating selected psychological and physiological conditions. Journal of Nervous and Mental Disease. 1995;183(7):478-84.

16. Taylor DN. Clinical and experimental evaluation of cranial TENS in the U.S.: A review. Acupuncture and Electro-Therapeutics Research. 1995;20(2):117-32.

17. De Felice EA. Cranial electrotherapy stimulation (CES) in the treatment of anxiety and other stress-related disorders: a review of controlled clinical trials. Stress medicine. 1997;13(1):31‐42.

18. Kirsch DL, Smith RB. The use of cranial electrotherapy stimulation in the management of chronic pain: A review. NeuroRehabilitation. 2000;14(2):85-94.

19. Gilula MF, Barach PR. Cranial electrotherapy stimulation: A safe neuromedical treatment for anxiety, depression, or insomnia [2]. Southern Medical Journal. 2004;97(12):1269-70.

20. Gilula MF, Kirsch DL. Cranial electrotherapy stimulation review: A safer alternative to psychopharmaceuticals in the treatment of depression. Journal of Neurotherapy. 2005;9(2):7-26.

21. Gilula MF. Cranial electrotherapy stimulation and fibromyalgia. Expert Review of Medical Devices. 2007;4(4):489-95.

22. Bystritsky A, Kerwin L, Feusner J. A pilot study of cranial electrotherapy stimulation for generalized anxiety disorder. Journal of Clinical Psychiatry. 2008;69(3):412-7.

23. Fitzgerald PB. The emerging use of brain stimulation treatments for psychiatric disorders. Australian and New Zealand Journal of Psychiatry. 2011;45(11):923-38.

24. Adeyemo BO, Simis M, Macea DD, Fregni F. Systematic review of parameters of stimulation, clinical trial design characteristics, and motor outcomes in non-invasive brain stimulation in stroke. Frontiers in Psychiatry. 2012;3(NOV).

25. Kirsch DL, Nichols F. Cranial Electrotherapy Stimulation for Treatment of Anxiety, Depression, and Insomnia. Psychiatric Clinics of North America. 2013;36(1):169-76.

26. Kavirajan HC, Lueck K, Chuang K. Alternating current cranial electrotherapy stimulation (CES) for depression. Cochrane Database of Systematic Reviews. 2014(7).

27. Marksberry J, Kirsch D, Nichols F, Price LR, Platoni KT. Efficacy of cranial electrotherapy stimulation for anxiety, PTSD, insomnia and depression: Military service members and veterans self reports. Brain Stimulation. 2015;8(2):311.

28. Trojak B, Sauvaget A, Fecteau S, Lalanne L, Chauvet-Gelinier JC, Koch S, et al. Outcome of non-invasive brain stimulation in substance use disorders: A review of randomized sham-controlled clinical trials. Journal of Neuropsychiatry and Clinical Neurosciences. 2017;29(2):105-18.

29. Jonas WB. Cranial electrical stimulation: What is it, and should we use it in practice? Annals of Internal Medicine. 2018;168(6):446-7.

30. Marksberry J. Benefits and harms of cranial electrical stimulation. Annals of Internal Medicine. 2018;169(4):268-9.

31. Shekelle PG, Cook IA, Miake-Lye IM, Booth MS, Beroes JM, Mak S. Benefits and harms of cranial electrical stimulation for chronic painful conditions, depression, anxiety, and insomnia a systematic review. Annals of Internal Medicine. 2018;168(6):414-21.

32. Ahmed MM, Sumner JM, Nissinen KJ, Haque S, Chau J, Diep M. NEUROIMAGING AND NEUROPSYCHOLOGICAL CHANGES IN ALZHEIMER'S DEMENTIA WITH TRANSCRANIAL MAGNETIC STIMULATION (TMS): CASE REPORT. Alzheimer's and Dementia. 2019;15(7):P259-P60.

33. Chalah MA, Ayache SS. Noninvasive brain stimulation and psychotherapy in anxiety and depressive disorders: A viewpoint. Brain Sciences. 2019;9(4).

34. Herrmann MJ, Mühlberger A, Ehlis AC, Deckert J, Polak T. A systematic review of non-invasive brain stimulation and fear extinction. Nervenheilkunde. 2019;38(8):537-41.

35. Marksberry J. The management of anxiety, insomnia and depression with cranial electrotherapy stimulation. Brain Stimulation. 2019;12(2):397.

36. Nuñez M, Zinbarg RE, Mittal VA. Efficacy and mechanisms of non-invasive brain stimulation to enhance exposure therapy: A review. Clinical Psychology Review. 2019;70:64-78.

37. Petrenko T, Retjunskiy K, Kublanov V, Petrenko A. Application of non-invasive electrostimulation of the autonomic nervous system in the complex treatment of children with psychosomatic disorders. Brain Stimulation. 2019;12(2):568-9.

38. Sagliano L, Atripaldi D, De Vita D, D'Olimpio F, Trojano L. Non-invasive brain stimulation in generalized anxiety disorder: A systematic review. Progress in Neuro-Psychopharmacology and Biological Psychiatry. 2019;93:31-8.

39. Vaschillo E, Vaschillo B, Buckman J, Bates M. New approach for brain stimulation. Brain Stimulation. 2019;12(2):393.

40. Vicario CM, Salehinejad MA, Felmingham K, Martino G, Nitsche MA. A systematic review on the therapeutic effectiveness of non-invasive brain stimulation for the treatment of anxiety disorders. Neuroscience and Biobehavioral Reviews. 2019;96:219-31.

41. Yosephi MH, Ehsani F, Daghiani M, Zoghi M, Jaberzadeh S. The effects of trans-cranial direct current stimulation intervention on fear: A systematic review of literature. Journal of Clinical Neuroscience. 2019;62:7-13.

42. Allida S, Cox KL, Hsieh CF, Lang H, House A, Hackett ML. Pharmacological, psychological, and non‐invasive brain stimulation interventions for treating depression after stroke. Cochrane Database of Systematic Reviews. 2020(1).

43. Freire RC, Cabrera-Abreu C, Milev R. Neurostimulation in anxiety disorders, post-traumatic stress disorder, and obsessive-compulsive disorder. 2020. p. 331-46.

44. Morriss R, Price L. Differential effects of cranial electrotherapy stimulation on changes in anxiety and depression symptoms over time in patients with generalized anxiety disorder. Journal of Affective Disorders. 2020;277:785-8.

45. Romanella SM, Sprugnoli G, Ruffini G, Seyedmadani K, Rossi S, Santarnecchi E. Noninvasive Brain Stimulation & Space Exploration: Opportunities and Challenges. Neuroscience and Biobehavioral Reviews. 2020;119:294-319.

46. Thibaut A, Shie VL, Ryan CM, Zafonte R, Ohrtman EA, Schneider JC, et al. A review of burn symptoms and potential novel neural targets for non-invasive brain stimulation for treatment of burn sequelae. Burns. 2020.

47. Yu B, Qiu H, Li J, Zhong C, Li J. Noninvasive Brain Stimulation Does Not Improve Neuropathic Pain in Individuals With Spinal Cord Injury: Evidence From a Meta-Analysis of 11 Randomized Controlled Trials. American journal of physical medicine & rehabilitation. 2020;99(9):811-20.

48. Barone M, Imaz F, Bordachar D, Ferreira I, Intelangelo L. Effect of pain neuroscience education and transcutaneous electrical nerve stimulation on trigeminal postherpetic neuralgia. A case report. Physiotherapy theory and practice. 2021:1-10.

49. Berkovitch L, Roméo B, Karila L, Gaillard R, Benyamina A. Efficacy of psychedelics in psychiatry, a systematic review of the literature. Encephale. 2021.

50. Brunyé TT, Patterson JE, Wooten T, Hussey EK. A Critical Review of Cranial Electrotherapy Stimulation for Neuromodulation in Clinical and Non-clinical Samples. Frontiers in Human Neuroscience. 2021;15.

51. Lefebvre-Demers M, Doyon N, Fecteau S. Non-invasive neuromodulation for tinnitus: A meta-analysis and modeling studies. Brain Stimulation. 2021;14(1):113-28.

**No outcome of interest(n=9)**

1. Kavirajan HC, Lueck K, Chuang K. Alternating current cranial electrotherapy stimulation (CES) for depression. Cochrane Database of Systematic Reviews. (7):CD010521.

2. Padjen AL, Dongier M, Malec T. Effects of cerebral electrical stimulation on alcoholism: a pilot study. Alcoholism: Clinical & Experimental Research.19(4):1004-10.

3. Pickworth WB, Fant RV, Butschky MF, Goffman AL, Henningfield JE. Evaluation of cranial electrostimulation therapy on short-term smoking cessation. Biological Psychiatry.42(2):116-21.

4. Morrish Jr RB. Suppression and prevention of the gag reflex with a TENS device during dental procedures. General dentistry. 1997;45(5):498-501.

5. Pickworth WB, Fant RV, Butschky MF, Goffman AL, Henningfield JE. Evaluation of cranial electrostimulation therapy on short-term smoking cessation. Biological psychiatry. 1997;42(2):116‐21.

6. Chu H, Li MH, Ye NY, Chiou WY. Effects of cranial electrostimulation on motion sickness induced by Coriolis stimulation. Journal of medical sciences (taipei, taiwan). 2012;32(2):69‐74.

7. Gense De Beaufort D, Sesay M, Stinus L, Thiebaut R, Auriacombe M, Dousset V. Cerebral blood flow modulation by transcutaneous cranial electrical stimulation with Limoge's current. Clinical Neurophysiology. 2013;124(10):e65.

8. Nct. A Single Blind Study of Cranial Electrical Stimulation in Bipolar II Disorder. https://clinicaltrialsgov/show/NCT01909011. 2013.

9. Tirado C, Washburn S, Covalin A, Khodaparast N. Use of transcutaneous auricular neurostimulation in the reduction of symptoms associated with opioid withdrawal. Neuromodulation. 2020;23(3):e322‐.

**Supplementary Table S3 Jadad scores**

| **First Author (year)** | **Randomisation** | **Blinding** | **Withdrawl and drop out** | | **Total scores** |
| --- | --- | --- | --- | --- | --- |
| **Barclay TH, 2014** | **2** | **2** | **1** | **5** | |
| **Gong BY, 2016** | **2** | **0** | **0** | **2** | |
| **Chen Y, 2007** | **1** | **0** | **0** | **1** | |
| **Do JK, 2021** | **2** | **2** | **1** | **5** | |
| **Cho S-Y, 2016** | **2** | **0** | **0** | **2** | |
| **Cork RC , 2004** | **1** | **2** | **0** | **3** | |
| **Lu L, 2014** | **1** | **1** | **0** | **2** | |
| **Wu WJ, 2020** | **1** | **2** | **0** | **3** | |
| **Tan G, 2011** | **2** | **2** | **0** | **4** | |
| **Lyon D, 2015** | **2** | **2** | **0** | **4** | |
| **NCT00723008** | **1** | **2** | **1** | **4** | |

**Supplementary Figure S4:** Risk of bias(Rob)


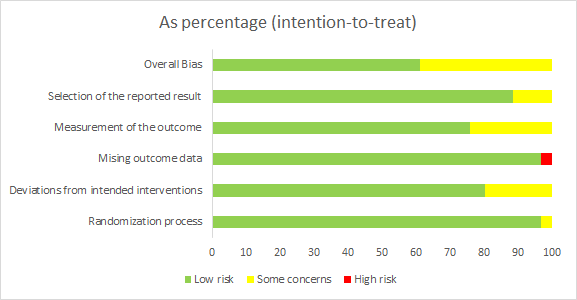


| Study ID | weight | domain1 | domain 2 | domain 3 | domain 4 | domain 5 | overall |
| --- | --- | --- | --- | --- | --- | --- | --- |
| Barclay TH, 2014 | 10.2 | low | Some concerns | low | low | Some concerns | Some concerns |
| Cho S-Y, 2016 | 1 | low | Some concerns | low | Some concerns | low | low |
| Cork RC, 2004 | 1 | low | low | low | low | low | low |
| Do JK, 2021 | 2.96 | low | low | low | low | low | low |
| Gong BY, 2016 | 9.25 | low | low | low | low | low | low |
| Lu L, 2014 | 13.88 | low | Some concerns | low | low | low | Some concerns |
| Lyon D, 2015 | 18.47 | low | low | low | low | low | low |
| NCT00723008 | 2.87 | low | Some concerns | low | low | low | Some concerns |
| Tan G, 2011 | 13.4 | low | low | low | low | low | low |
| Wu WJ, 2020 | 1 | low | low | low | low | low | low |
| Chen Y, 2007 | 1 | low | low | low | Some concerns | low | Some concerns |

Domain 1: Randomization process

Domain 2: Deviation from the intended intervention

Domain 3: Missing outcome data

Domain 4: Measurement of the outcome

Domain 5: Selection of the reported result

**Supplementary Table S5A:** Random-effects meta-regression analyses of potential moderators of the association of CES and anxiety symptoms

| **Clinical variables** | **Number of datasets** | **coefficient; 95% CI** | ***P*-value** |
| --- | --- | --- | --- |
| Female (%) | 11 | -0.7809;-2.9593 to 1.3975 | 0.4823 |
| Jadad score | 11 | -0.1012;-0.5454 to 0.3431 | 0.6553 |
| total sessions | 11 | 0.0056;-0.0120 to 0.0233 | 0.5316 |
| duration of each sessions | 11 | -0.0063;0.0356 to 0.0231 | 0.6758 |

* *p*<0.05

**Supplementary Table S5B:** Random-effects meta-regression analyses of potential moderators of the association of CES and depression symptoms

| **Clinical variables** | **Number of datasets** | **coefficient; 95% CI** | ***P*-value** |
| --- | --- | --- | --- |
| Female (%) | 8 | 0.4252;-1.6720 to 2.5224 | 0.8486 |
| Jadad score | 8 | -0.2173;-0.7718 to 0.3373 | 0.7954 |
| total sessions | 8 | -0.0011;-0.0282 to 0.0381 | 0.7704 |
| duration of each sessions | 8 | 0.0025;-0.0727 to 0.0777 | 0.9478 |

* *p*<0.05

**SupplementaryTable S6** Drop-out and adverse event

| Author, year, study design | Drop-out (N/baseline sample, %) | Adverse event (description: N) |
| --- | --- | --- |
| Barclay TH, 2014, double blind RCT  Anxiety disorder | Intervention group: 3/60=5%  Control group: 4/55=7.2% | No adverse events reported: 0 |
| Gong BY, 2016, RCT  Functional constipation ‘ s anxiety symptoms | Intervention group: 14/38=36.8%  Control group: 16/36=44.4% | Both group  pulsing, tickling, or tingling sensations of the ears : 3  ear clips too tight : 2  drowsiness : 1 |
| Chen Y, 2007,RCT, mixed anxiety and depressive disorder | Intervention group: 0/30=0%  Control group: 0/30= 0% | Not mentioned |
| Do JK, 2021,  double blinded RCT  Headache ,anxiety symptoms | Intervention group: 1/12: 8.3%  Control group: 1/12= 8.3% | Not mentioned |
| Cho S-Y, 2016,RCT,Obessity patient,anxiety symptoms | Both group: 0% | Not mentioned |
| Cork RC, 2004,double blinde RCT  Fibromyalgia,anxiety symptoms | Not mentioned | Not mentioned |
| Lu L, 2014,RCT,anxiety disorder ,anxiety symptoms | Intervention group: 0/60=0%  Control group: 0/60= 0% | Not mentioned |
| Wu WJ, 2020, double blinded RCT,  Tic disorder ,anxiety symptoms | Intervention group: 3/32 = 9.4%  Control group: 6/30= 20% | Intervention group: 18  Ear discomfort:5 ,headache: 2,dizzy: 5,sleepy: 2 ,nausea:1, Tinnitus:2  Control group: 12  Ear discomfort:6 ,headache:1,dizzy:1,sleepy: 0 ,nausea:1, Tinnitus:3 |
| Tan G, 2011,double blind RCT  Spinal cord injury, anxiety symptoms | Intervention group: 1/46 = 2.2%  Control group: 4/59= 6.8% | Intervention group:32  Ear disconfort:12,legs and buttock discomfort:3,tinnutis:1,sleepy:7,dizzy:3, Nause:1,headache:2, unusual taste of mouth:1,increased pain:2  Control group:25  Ear disconfort:6,legs and buttock discomfort:3,tinnutis:1,sleepy:4,dizzy:1  Nause:2,headache:4, unusual taste of mouth:1, palpitation and chest tightness:2,increased pain:1 |
| Lyon D, 2015, double blinded RCT,Breast cancer,anxiety symptoms | Intervention group: 14/84 = 16.7%  Control group:16/83= 19.2% | Intervention group: 1 has seizure  Control group:0 |
| NCT00723008 | Intervention group: 5/10=50%  Control group: 5/9=55.6% | Intervention group:9  Nausea: 1, ear pain:3 , headache:1 , Increased Injury Site Pain:4 ,increased irritability : 1, night terrors:1 ,insomnia:2  Control group:8  stiffness: 1, ear pain:4 , headache:0 , Increased Injury Site Pain:3 , Lightheadedness: 1,increased irritability : 0, night terrors:1 ,insomnia:2 |

**Supplementary Figure S7:** Drop-out rate and adverse events rate

Drop-out rate


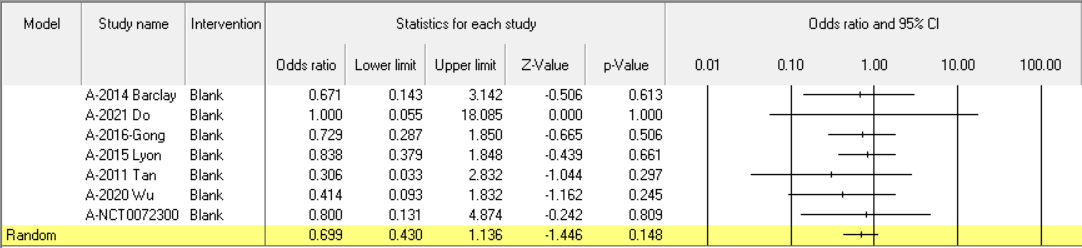

Supplement: Supplementary file 1 [file Data_Sheet_1.docx]
